# TreeKnit: Inferring ancestral reassortment graphs of influenza viruses

**DOI:** 10.1371/journal.pcbi.1010394

**Published:** 2022-08-19

**Authors:** Pierre Barrat-Charlaix, Timothy G. Vaughan, Richard A. Neher

**Affiliations:** 1 Biozentrum, Universität Basel, Basel, Switzerland; 2 Swiss Institute of Bioinformatics, Basel, Switzerland; 3 ETH Zurich, Department of Biosystems Science and Engineering, Basel, Switzerland; International Center for Theoretical Physics - South American Institute for Fundamental Research, BRAZIL

## Abstract

When two influenza viruses co-infect the same cell, they can exchange genome segments in a process known as reassortment. Reassortment is an important source of genetic diversity and is known to have been involved in the emergence of most pandemic influenza strains. However, because of the difficulty in identifying reassortment events from viral sequence data, little is known about their role in the evolution of the seasonal influenza viruses. Here we introduce TreeKnit, a method that infers ancestral reassortment graphs (ARG) from two segment trees. It is based on topological differences between trees, and proceeds in a greedy fashion by finding regions that are compatible in the two trees. Using simulated genealogies with reassortments, we show that TreeKnit performs well in a wide range of settings and that it is as accurate as a more principled bayesian method, while being orders of magnitude faster. Finally, we show that it is possible to use the inferred ARG to better resolve segment trees and to construct more informative visualizations of reassortments.

## Author summary

Influenza viruses evolve quickly and escape immune defenses which requires frequent update of vaccines. Understanding this evolution is key to an effective public health response. The genome of influenza viruses is made up of 8 pieces called segments, each coding for different viral proteins. Within each segment, evolution is an asexual process in which genetic diversity is generated by mutations. But influenza also diversifies through reassortment which can occur when two different viruses infect the same cell: offsprings can then contain a combination of segments from both viruses. Reassortment is akin to sexual reproduction and can generate viruses that combine segments from diverged viral lineages. Reassortment is a crucial component of viral evolution, but it is challenging to reconstruct where reassortments happened and which segments share history. Here, we develop a method called TreeKnit to detect reassortment events. TreeKnit is based on genealogical trees of single segments that can be reconstructed using standard bioinformatics tools. Inconsistencies between these trees are then used as signs of reassortment. We show that TreeKnit is as accurate as other recent methods, but runs much faster. Our method will facilitate the study of reassortment and its consequences for influenza evolution.

This is a *PLOS Computational Biology* Methods paper.

## Introduction

Influenza viruses evolve rapidly and change their surface proteins, which allows them to evade preexisting immunity and reinfect their hosts. The viral genome is made of 8 RNA segments that encode for 11 different proteins, with segments coding for the surface proteins haemagglutinin (HA) and neuraminidase (NA) being the most important for immune escape. In each segment, evolution is an asexual process in which diversity is generated by mutations. However, when a host cell is simultaneously infected by more than one virus, offspring viruses can carry segments from several parents—a process known as reassortment [1]. Genomic reassortment is akin to sexual reproduction and can generate viruses with novel genetic constellations. In particular, it has been found to be the cause of most pandemic influenza strains [2, 3].

**Fig 1:**
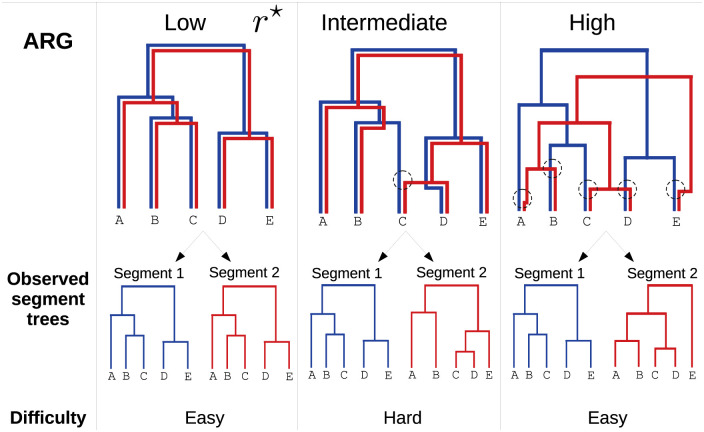
Example of ARGs for five sampled strains and for two segments (blue and red). Reassortments are shown as black circles in each ARG. Based on the scaled reassortment rate in the population *r*^⋆^, three regimes can be identified. **Left**: Very low reassortment rate. Reassortments are very rare, and every strain inherits its two segments from the same parent. The ARG is equal to the gene trees, and reconstructing it is easy. **Center**: Intermediate reassortment rate. Some exchange of segments takes place: some strains do not inherit their segments from the same parent (here, strain C). The segments trees have different topologies, but are still relatively similar. Inferring the position of reassortments from the gene trees is non trivial. **Right**: Very high reassortment rate. A reassortment takes place on every branch of the ARG before the first coalescence. The two segments have independent evolutionary histories, and the segment trees share no structure. Inference of reassortments becomes easy again.

The genealogy of a single segment is described by a tree, whose leaves correspond to observed sequences and internal nodes to the ancestry of different lineages. Many methods exist to reconstruct this tree from gene sequences [4–6]. However, trees are not well suited to describe genealogies of full genomes in the presence of reassortment since lineages can then have different ancestors for their different segments. A more adapted concept is the so-called Ancestral Reassortment Graph (ARG), or Ancestral Recombination Graph in the context of recombination. Internal nodes of the ARG represent either coalescence of different lineages, in which case they have a unique ancestor as internal tree nodes, or the emergence of a new lineage from a reassortment, in which case they may have several ancestors. Simple examples of ARGs for two segments are shown in Fig 1.

The knowledge of the ARG for a set of influenza sequences would be of major interest, mainly as it would shine light on the role of reassortment in the evolution of influenza. While the importance of major reassortment events in the formation of pandemic strains is known, much less can be said on the effects of smaller scale intra flu-lineage reassortments. Several studies have tackled this problem, with significant discrepancy in their results [7–9]. This is likely due to the lack of a robust and efficient method to infer the whole set of reassortments in the history of a large and representative set of viral genomes. In addition, knowing the ARG would allow a more accurate reconstruction of tree branch lengths in regions without reassortments by using the sequences of several segments, or a better visualization of pairs of trees of different segments by disentangling tanglegrams as much as possible (see S1 Fig).

A common method to identify reassortments is to reconstruct segment trees and manually compare them [10, 11], which is time consuming and error prone. A number of automated methods have also been developed. Some do not go through the step of reconstructing phylogenies and instead compare the sequence distance of different strains for different segments [12, 13]. Other approaches consist in finding discrepancies between segment trees, either using topology [14–16] or mutation patterns on the branches of the trees [8]. Sets of probable reassortments are then deduced from these discrepancies, typically using a confidence score. A common point to all these methods is that they only identify a subset of the reassortments that occurred in the genealogy, which could result in a mis-interpretation of the importance of reassortment. Additionally, it is not possible to fully reconstruct the genealogy since differences between trees remain even after accounting for the inferred reassortments. Recently, a method for directly inferring the ARG from sequences has been proposed that extends the principles used to infer phylogenetic trees to data containing reassortments [9, 17]. It does so by using a coalescence-reassortment model to assign a probability to any ARG based on observed sequences and then samples from this probability. While this model based approach is appealing, it is computationally expensive and limited to medium size datasets.

Here we propose TreeKnit, a fast method to infer ARGs from pairs of segment trees that processes by “knitting” the trees together starting from the leaves. The underlying idea is that topological differences between trees are caused by reassortments, and that we can thus introduce reassortments so as to minimize these incompatibilities. In a first part, we describe how TreeKnit works. We then estimate its performance and limitations on simulated genealogies and show that it can be used to better resolve segment trees. Finally, we compare it to two existing methods to infer reassortments in influenza genealogies.

## Methods

Whether inferring ARGs is easy or hard and whether it is useful or not depends on the relative strength of coalescence and reassortment. Qualitatively, we can distinguish three main regimes represented in Fig 1. For a very low reassortment rate, reassortments are so rare that the ARG can be considered tree-like, with the two segment trees being identical. Recovering the ARG from the knowledge of the segment trees is then trivial. On the contrary, for a very large reassortment rate, the first reassortments along a lineage occur well before any pair of strains coalesce to a common ancestor. The two segments evolve in practice independently, and their trees have no shared structure. Coalescences between different segments and additional reassortments can occur deeper in the ARG, but would only connect to observed sequences by branches that are not shared by the two segment trees (see S3 Fig for an illustration). As they leave almost no trace on the segment trees, these deeper events cannot be identified by the method presented here, and we will ignore them in the following. Inferring the observable part of the ARG is again easy although uninformative: one only has to introduce a reassortment above each leaf. The intermediate case is both the hardest and the most interesting one. Indeed, reassortments are then rare enough that the segment trees share a lot of structure, but sufficiently frequent for the problem to not be trivial.

### Maximally Compatible Clades (MCC)

A central concept for our method are *Maximally Compatible Clades (MCCs)*. Given a two segment ARG that embeds two trees, one obtains the MCCs by removing all branches that correspond to only one tree, and keeping those that are common to both. In Fig 1, this would amount to removing all branches that have only one color and keeping those that are both red and blue. This operation results in a set of disjoint trees, each of those being one MCC. Note that MCCs are not necessarily clades in the segment trees, since they can be nested. It is convenient to refer to an MCC by the leaves that it contains, and we will do so in the following.

MCCs have several properties that makes them a very useful concept for thinking about ARGs: (i) If both segment trees *and* all MCCs are known, so is the observable part of the corresponding ARG. This follows from the fact that MCCs are the regions where the two trees are “stitched” together in the ancestral graph. Our method reconstructs ARGs using this idea and is effectively a method to *infer MCCs given a pair of trees*. Once the MCCs are known, the only informations missing to fully reconstruct the observable ARG are the times at which reassortments occurred on internal branches. (ii) There is a one-to-one correspondence between MCCs and observable reassortment events. This is a simple consequence of the fact that reassortments correspond to the separation of lineages of different segments in the ARG, and therefore to the transition between a region where branches are common to both trees to a region where they are not. By definition the root of an MCC must be located right where this separation occurs. The only exception to this rule is the case of an MCC that contains the root of one of the segment trees. The implication is that the number of reassortments in the genealogy of two segments is equal to the number of MCCs in their ARG, minus one if one of the MCCs contains a root. (iii) Restricting segment trees to an MCC results in two subtrees with the same topology. MCCs are maximal in the sense that extending them by adding nodes results in *topologically different* subtrees in the two segments. This last property is important for our method: finding MCCs is equivalent to finding maximal sets of leaves that give rise to subtrees with matching topologies.

### Finding MCCs: The TreeKnit method

**Fig 2:**
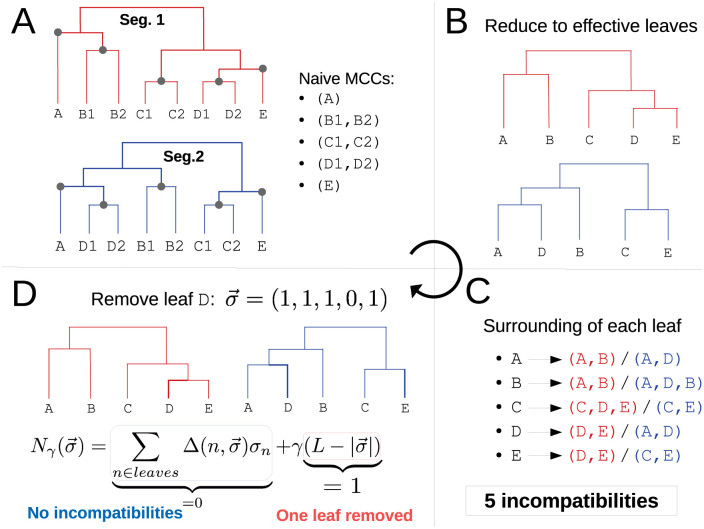
Schematic of the iterative algorithm. **A**: Construction of the naive MCCs. Circles indicate the root of the five clades that match exactly in the two trees (slightly highlighted branches). Trying to grow one of these clades gives inconsistent results in the two trees: *e.g*. growing the MCC (B1,B2) gives clade (A,B1,B2) in the first tree and (A,D1,D2,B1,B2) in the second. **B**: Trees obtained after reducing trees of **A** to their naive MCCs: each clade is represented by a single effective leaf. **C**: Counting incompatibilities in the reduced trees. For each effective leaf, the clades defined by its direct ancestor in the two trees are compared, and each mismatch counts as one incompatibility. **D**: Enforcing reassortments on some leaves to remove incompatibilities. A configuration $\vec{\sigma }$ is associated to each set of removed leaves. The scoring function $N_{\gamma }\left(\vec{\sigma }\right)$ adds the number of remaining incompatibilities given $\vec{\sigma }$ and the number of removed leaves multiplied by *γ*. The optimal set of reassortments is found by minimizing $N_{\gamma }\left(\vec{\sigma }\right)$, *e.g*. removing D is optimal if *γ* < 5.

The input to TreeKnit are two rooted segment trees that share leaves. TreeKnit is an iterative method that alternates between trying to grow a set of compatible clades (CCs) until they are maximal, and identifying reassortment events that prevent further expansion of these compatible clades. At the start of the algorithm, the CCs are simply the leaves of the trees. The iterative cycle is illustrated in Fig 2 and consists of the following four steps.

(i) Perform a naive maximization of compatible clades (CCs): grow CCs by adding internal nodes as long as the obtained clades are exactly equivalent in the two trees (Fig 2**A**). The resulting compatible regions are called the naive MCCs, where the “maximal” term will be justified in the next section.
(ii) Collapse the naive MCCs into effective nodes (Fig 2**B**). This allows us to ignore their topological details. Note that if we applied step *(i)* to the reduced trees, we would find naive MCCs consisting only of leaves, by construction.
(iii) Count topological incompatibilities in the reduced trees (Fig 2**C**). For each leaf, compare its surroundings in the two trees, and count one incompatibility if the two surroundings do not match. The surrounding of a leaf is defined as the clade defined by its parent node.
(iv) Enforce reassortments on some leaves in order to *minimize* the number of incompatibilities (Fig 2**D**). Effective leaves above which a reassortment is enforced are *removed from the trees*, which reduces the number of incompatibilities. The cost associated with removing a leaf is determined by parameter *γ*. Effective leaves removed at the end of this operation are added to the list of final MCCs. If no leaf was removed as a result of the optimization, *i.e*. if no reassortment was enforced, stop here. Otherwise, go back to step *(i)*

A leaf *n* can give rise to an incompatibility counted in step *(iii)* for two reasons: if a reassortment took place on the branch leading to *n*, it will be observed in different regions in the two trees, corresponding to the two ancestral viruses that took part in the reassortment; or if *n* is “close” to another reassorted leaf in one of the trees and not in the other, in which case it will also have different surroundings. In the example of Fig 2, D corresponds to the first situation, and A,B,C and E to the second.

Given two reduced trees of *L* effective leaves after step *(ii)*, the idea of step *(iv)* is to reduce as much as possible the number of topological incompatibilities by removing (mark as reassortants) a minimal number of leaves. We encode the current state of each leaf *n* in the binary variable *σ*_*n*_ ∈ {0, 1}: it is set to 1 if leaf *n* is present in the tree, and to 0 if it is removed from the tree. Furthermore, we define $\Delta (n,\vec{\sigma })=0$ if the trial configuration $\vec{\sigma }$ resolves the incompatibility above node *n* (compare Fig 2**C** and 2**D**), and $\Delta (n,\vec{\sigma })=1$ otherwise. For any combination of removed leaves $\vec{\sigma }$, we now define the scoring function $N_{\gamma }\left(\vec{\sigma }\right)$:

$$\begin{array}{c} N_{\gamma }\left(\vec{\sigma }\right)=\sum_{n\in \text{leaves}}\Delta \left(n,\vec{\sigma }\right)\sigma_{n}+\gamma (L-|\vec{\sigma }\left|\right), \end{array}$$ (1)

where $|\vec{\sigma }|$ is the *l*1-norm of $\vec{\sigma }$. $N_{\gamma }\left(\vec{\sigma }\right)$ is composed of two terms with a simple interpretation: the first sums over leaves and counts the number of remaining incompatibilities, while the second counts the number of removed leaves with a removal cost *γ*.

The optimal set of leaves to remove is then found by finding the configuration ${\vec{\sigma }}^{⋆}$ that minimizes *N*_*γ*_. As this function can have several local and global minima, minimization is performed efficiently by simulated annealing [18], see S1 Text. In the case of Fig 2**D** and for *γ* ≤ 5, the optimal configuration is the one that removes leaf D only, that is ${\vec{\sigma }}^{⋆}=\left(1\;1\;1\;0\;1\right)$, with a score $N_{\gamma }\left({\vec{\sigma }}^{⋆}\right)=\gamma$. Removing any single other leaf gives a score 4 + *γ*, and is thus always worse than removing D. Not removing any leaf gives a score 5, which becomes optimal if *γ* > 5.

The parameter *γ* plays a key role in the result of the method and thus on the reconstruction of the ARG. In the next paragraphs, we discuss the behavior of TreeKnit for extreme values of *γ* and explain some underlying ideas.

### Naive MCCs: *γ* → ∞

If *γ* is very large, *i.e*. of the order of the number of leaves in the reduced trees, removing leaves in step *(iv)* has a prohibitive cost. The MCCs returned by the algorithm will thus be the ones found in step *(i)*, that is the *naive MCCs*.

This *n*aive approach has a simple interpretation: any two clades with the exact same topology in two trees are matched, but compatible regions are not extended further. The limit can thus be thought of as a *conservative* approach to the reconstruction of MCCs that avoid over-extending MCCs.

On the other hand, since there is a one to one mapping between MCCs and inferred reassortments, the naive method tends to over-estimate the number of reassortments. This evident in Fig 2: five naive MCCs are found, corresponding to five inferred reassortments since none of them contains the root of one of the trees. The naive method introduces a reassortment for each incompatibility it finds and treats the two types of incompatibilities discussed above identically. However, to infer a more accurate set of MCCs, it is necessary to identify the first type of incompatibility corresponding to a reassortment along the branch leading to the leaf. After identifying these and removing the corresponding MCC, other incompatibilities tend to resolve as is the case in the example in Fig 2: A single reassortment above clade (D1, D2) explains the differences between the two trees.

### Approximately parsimonious MCCs: *γ* = 1

We have seen above that every incompatibility gives rise to one inferred reassortment in the naive approach. On the other hand, we also know that an incompatibility above one leaf might not be due to a reassortment above it, but rather to its proximity to another reassorted leaf. This means that if we observe *N* incompatibilities, we can often resolve them by using less than *N* reassortments.

For *γ* = 1, the scoring function *N*_*γ*_ of Eq 1 has a simple interpretation in terms of *parsimony*. Its first term counts the number of remaining incompatibilities, approximating the number of remaining reassortments predicted by the naive approach once the leaves with *σ*_*n*_ = 0 have been removed. The other term $\left(L-|\vec{\sigma }|\right)$ counts the number of removed leaves, which are *enforced* reassortments. $N_{1}\left(\vec{\sigma }\right)$ thus approximates the total number of reassortments for a configuration $\vec{\sigma }$. Since the configuration ${\vec{\sigma }}^{⋆}$ minimizes *N*_1_, it can be interpreted as a parsimonious explanation of the topological differences between the trees.

### Bridging parsimonious and naive approaches: Intermediate *γ*

Fig 2 shows an example where a parsimonious method clearly outperforms the naive one. In some cases, for example the high reassortment rate case shown on the right panel of Fig 1, this is not true: in this case the correct MCCs consist of single leaves, and the naive approach then performs well. However, it is possible to explain the tree with fewer reassortments. Removing leaves A,B and C, corresponding to configuration $\vec{\sigma }=\left(0\;0\;0\;1\;1\right)$, will result in $N_{1}\left(\vec{\sigma }\right)=3$, since the two remaining leaves D and E will then form a compatible clade in the two trees (three enforced reassortments, no incompatibility left). More generally, given an ARG of *L* leaves and with an infinitely high reassortment rate, it is always possible for the pseudo-parsimonious approach to enforce reassortments on *L* − 2 leaves, and have the remaining 2 form a compatible clade, thus obtaining *L* − 2 reassortments in total, instead of *L*. This is not surprising, as it is expected that a parsimonious method performs poorly when there are many reassortments.

Parameter *γ* in Eq 1 tunes the “aggressiveness” with which the algorithm tries to grow compatible clades. In the pseudo-parsimonious approach, *N*_*γ*_ stays constant if one enforced reassortment “fixes” exactly one incompatibility. On the other hand, if *γ* > 1, every removed leaf must fix more than one incompatibility for *N*_*γ*_ to stay constant. As a consequence, it is harder to remove leaves and thus to grow MCCs. In the extreme limit of *γ* → ∞, MCCs cannot be grown and we fall back to the naive method.

**Fig 3:**
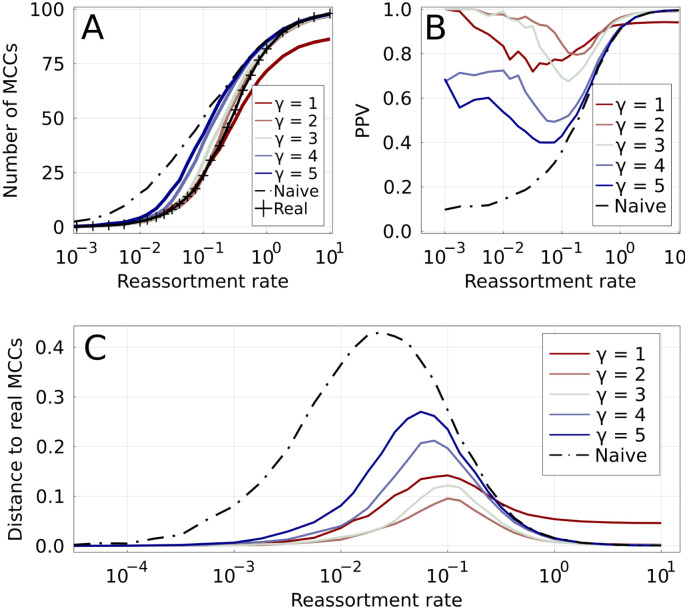
Accuracy of inferred MCCs in simulated ARGs. Increasing values of *γ* are shown by colored lines, from red to blue. **A**: Number of MCCs found by different methods as a function of the reassortment rate. The real number of MCCs is represented by the marked black line. The naive method (dashed black line) overestimates the number of MCCs for low *r*^⋆^, while the parsimonious one (*γ* = 1) underestimates it for high *r*^⋆^. **B**: Positive predictive value for reassortments: fraction of inferred reassortments that are indeed present in the real ARG. The low number of reassortments results in a relatively large uncertainty for this quantity for *r*^⋆^ ≪ 1. **C**: Distance between inferred and real MCCs for different methods. The distance is based on the variation of information [20].

The parameter *γ* thus allows us to interpolate between pseudo-parsimonious and naive approaches, and can be thought of as how “conservative” the inference of MCCs is. Note that given the discrete nature of Eq (1), the sharpest changes of behavior of *N*_*γ*_ happen when *γ* crosses an integer value. In the following, we will mostly use integer values *γ* (comp. Fig 3).

### Poorly resolved trees

**Fig 4:**
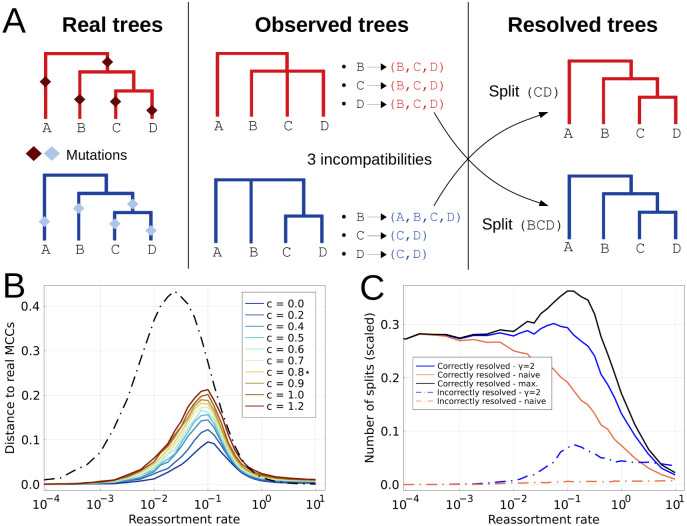
Effect of poorly resolved trees on the inference of MCCs. **A**: Pre-resolving trees before inferring MCCs. The approach is greedy: every split of one tree that is compatible with the other is introduced in the other. **B**: VI distance to real MCCs as a function of *r*^⋆^ for different tree resolutions *c*, using *γ* = 2. The dashed line corresponds to the naive method *γ* → ∞. The quality of the inference decreases with *c*. *c*^⋆^ ≃ 0.8 corresponds to levels found in A/H3N2 influenza trees with strains from the same season. **C**: Quality of the resolution of trees *after* having inferred MCCs. This combines splits introduced by the pre-resolution step, and splits known once the MCCs are inferred. The number of correctly inferred splits is shown, scaled by the number of splits that would be necessary to make the trees binary. The black line indicates the performance if the MCCs were exactly known.

Trees inferred from sequences are often not completely resolved: branches in the actual genealogy along which no mutations happened will not appear in the reconstructed tree. This results in polytomies or multifurcations: internal nodes with more than two offspring. On a branch of the ARG shared by the two segment trees, it is possible that mutations occurred in one of the segments and not in the other, so that a polytomy will be present in one reconstructed segment tree and not in the other. Fig 4**A** gives an example of such a situation.

If the polytomies are in parts of the segment trees shared in the ARG, one can improve phylogenetic resolution by using the sequences of both segments to infer topology and branch length. However, these shared parts of the ARG need to be identified first, which in turn is hampered by lack of phylogenetic resolution.

In order to overcome this problem, it is necessary to disentangle topological incompatibilities that are due to reassortment from those due to lack of resolution. We reduce the latter by resolving polytomies in each tree using the clades observed in the other. Formally, given two trees $T_{1}$ and $T_{2}$, we introduce in $T_{1}$ every split of $T_{2}$ that is compatible with the set of splits in $T_{1}$, and inversely. This assumes that topological differences that could be trivially explained by lack of phylogenetic resolution are never due to reassortment.

The right panel of Fig 4**A** sketches this resolution procedure. In this simple example, the resolving procedure allows one to recover perfect binary trees. However, it is important to note that many incompatibilities due to polytomies are not found in this way. This is especially true when the several descendants of a large polytomy are involved in reassortment events either within the clade or with other parts of the population. Situations where large polytomies and reassortments are entangled in the same clades also represent a challenge to the optimization procedure, as they tend to create local minima in the *N*_*γ*_ function. Indeed, in these cases it is often necessary to remove several leaves (i.e. identify as recombinants) from the polytomy before incompatibilities are fixed and the MCCs can be extended. Overall, poor resolution represents a loss of information regarding the genealogies, and makes the problem of finding reassortments intrinsically harder.

### Implementation and code availability

The code of TreeKnit is available at https://github.com/PierreBarrat/TreeKnit. It is implemented in Julia, and also provides a simple CLI script that returns an ARG file as an extended Newick string [19]. Other codes that were used for this work are listed here:

- The implementation for the simulation of ARGs can be found at https://github.com/PierreBarrat/ARGTools
- Miscellaneous functions that were used to evaluate the performance of TreeKnit are available at https://github.com/PierreBarrat/TestRecombTools.

The input to TreeKnit are two segment trees in the form of two Newick files. Requirements on the trees are the following:

- The trees must share all leaf nodes.
- Each tree must represent the genealogy of a genomic unit that does not experience reassortment/recombination. This is the case for trees of individual genome segments of influenza viruses.
- The two trees must be rooted in a consistent way. For instance, one can use the same outgroup to root both.
- Because TreeKnit fully trusts the topology of input trees (more below), all internal nodes should have a strong support. Low support internal nodes can cause TreeKnit to infer spurious reassortments. We highly recommend to remove any internal branch that is not supported by at least one mutation. In addition, we found that removing every internal node with a bootstrap value inferior to 75 gives robust results, see S4 Fig.

For simplicity, TreeKnit only takes a pair of trees as input and ignores the sequences that were used to build them. For this reason, it fully trusts the topology of these input trees: the internal nodes are taken as hard topological constraints regardless of the branch length or of confidence values. Since TreeKnit introduces reassortments to explain topological incompatibilities, uncertainty about internal nodes directly translates into uncertainty in the inferred reassortments. For this reason, it is generally preferable to remove internal nodes with low support from the trees, effectively transforming topological constraints into polytomies. Overall, the philosophy of TreeKnit is that the problem of poorly supported nodes should be dealt with before passing the trees to the algorithm.

TreeKnit expects the branch length of the trees to represent an average number of mutations per sequence position. However, this information is not crucial to the function of the algorithm (see S1 Text).

The output of TreeKnit is:

- A text file containing the list of inferred MCCs defined by their leaves.
- An ARG representing the evolution of the two input segments, written using the extended Newick format [19].
- Trees obtained by resolving each input segment tree using the other one and the knowledge of MCCs.
- A text file mapping internal nodes of the ARG to internal nodes of each resolved tree, whenever possible.

## Results

### Validation on simulated genealogies

We simulated two segment ARGs of one hundred leaves using a coalescent-reassortment process for different values of a scaled reassortment rate *r*^⋆^: coalescence dominates reassortment when *r*^⋆^ ≪ 1, and reassortment dominates coalescence when *r*^⋆^ ≫ 1. Details of the simulations can be found in S1 Text. Individual segment trees are then extracted from simulated ARGs, and we use TreeKnit to infer MCCs and compare them to the ground truth.

Fig 3**A** shows the number of MCCs *N*_*mcc*_, both real and inferred, as a function of *r*^⋆^. Note that by definition, each MCC corresponds to one reassortment in the ARG, except if it contains the root of one of the trees. Therefore, the number of reassortments is equal to either *N*_*mcc*_ or *N*_*mcc*_ − 1. As expected, the real number of MCCs (marked black line on the figure) is 1 for very low reassortment rates, and equal to the number of leaves for very high reassortment rates (see Fig 1 for an example).

At fixed *r*^⋆^, the number of inferred MCCs varies systematically with the parameter *γ*. The naive method (*γ* → ∞) is by construction conservative when merging MCCs and consistently overestimates the number of MCCs for *r*^⋆^ ≲ 1 (see discussion in the Methods section). In contrast, the parsimonious method (*γ* = 1) estimates the number of reassortments for low *r*^⋆^ accurately, but clearly underestimates it for *r*^⋆^ ≳ 0.5. Intermediate values of *γ* fall between these two extremes, with *γ* = 2 and *γ* = 3 being particularly close to ground truth.

Inferring the correct number of MCCs does not necessarily imply that they are correct. Fig 3 shows the positive predictive value (PPV), the fraction of correctly predicted reassortments, as a function of *r*^⋆^. For *γ* equal 2 or 3, the three regimes discussed on Fig 1 are immediately visible: inference is trivially easy in the regions of high and low *r*^⋆^, and the PPV is then close to one. For *γ* = 1, the PPV plateaus below one for high *r*^⋆^ meaning that TreeKnit not only infers too few reassortments, but some of those inferred are incorrect. ARG inference is hardest at intermediate reassortment rates. But even in this region the PPV is above 70% for *γ* = 1 and *γ* = 3, and above 80% for *γ* = 2. This suggests that reassortments are predicted with good accuracy by our method if *γ* is chosen in the right range of values. High values of *γ* overestimate the number of reassortment events at low *r*^⋆^, resulting in a low *PPV*.

The fraction of correctly predicted reassortments is only a limited measure of how well the ARG is recovered as all events, whether deep in the tree or on a terminal branch, are weighted equally. We propose an alternative measure to globally assess the accuracy at which the inference represents the truth based on the idea that MCCs define a *clustering* of leaves. Two clusterings of the same set can be compared using the variation of information (VI) [20]. The VI of two partitions of a set is equal to the difference between the sum of the entropies of the two partitions and their mutual information times two. It is 0 if the two partitions are identical, and equal to log(*L*) if the two partitions are maximally different, where *L* is the number of elements of the set, here the number of leaves in the trees. Here, we scale the VI by log(*L*) so that it varies between 0 and 1. While the values of VI are not as directly interpreted as a fraction of true positives, they constitute a global measure of how close a set of inferred MCCs is to the ground truth and can thus be used to compare methods.

Fig 3**C** shows the VI distance between the real MCCs and the ones inferred by TreeKnit for different *γ*. Regions where *r*^⋆^ ≫ 1 and *r*^⋆^ ≪ 1 result in accurate inference for *γ* > 1. As expected, the performance of the naive method starts to decrease as soon as *r*^⋆^ rises above very small values, or in other words as soon as reassortments appear in the ARG. As before, *γ* = 2 seems to be an optimal value across the entire range of reassortment rate. In S1 Text, we show that *γ* = 2 to 3 is optimal for different coalescent processes (S5 and S6 Figs), as well as for ARGs with asynchronously sampled leaves (S7 Fig). Therefore, fine-tuning of *γ* does not seem necessary.

Another way of estimating the accuracy of the inference is to look at predictions made for individual branches of the segment trees. For one of the trees, each branch can either be predicted to be shared with the other tree in the ARG, or to be specific to its tree (see illustration in Fig 1). S8 Fig shows the accuracy at which individual branches are predicted to be shared or not for different values of *γ*. As expected, *γ* = 1 results in branches incorrectly predicted to be shared, while for large values of *γ* shared branches are often missed. The values of *γ* lying in between smoothly interpolate between these two behaviors. *γ* can thus be seen as a way to balance between different types of false predictions for branches of the ARG.

### Increasing phylogenetic resolution of segment trees

While simulated trees are completely binary, trees reconstructed from genome sequences often lack resolution resulting in polytomies. This problem is particularly acute when the sample consists of many closely related viruses. As stated in the Methods section, poor resolution of trees leads to complications for our topology based method: topological differences due to reassortment must be disentangled from those due to different polytomies in segment trees, see Fig 4**A** for an example. Polytomies are thus a source of errors in the inference of MCCs. At the same time, the knowledge of the MCCs allows us to better resolve trees: in regions of shared branches in the ARG, the two trees must have the same splits, *i.e*. the same internal nodes. This means that once the MCCs are inferred, it is possible to introduce some of the splits of each tree in the other, and therefore to partly resolve them.

To quantify the errors in ARG reconstruction due to limited resolution and to assess the quality of the resolution once the MCCs are known, we simulated ARGs with limited resolution (see S1 Text). A parameter *c* ≥ 0 controls the amount of polytomies: *c* plays the role of an inverse mutations rate and *c* = 0 corresponds to perfectly resolved binary trees where every branch is supported by mutations, while *c* → ∞ corresponds to completely unresolved star-trees. A value of *c*^⋆^ ≃ 0.8 results in a level of resolution that is quantitatively close to the one observed in trees of A/H3N2 HA with strains of the same season (see S9 and S10 Figs).

Fig 4**B** shows the VI distance of inferred MCCs to the real ones (using *γ* = 2) as a function of *r*^⋆^ for varying resolution of the segment trees controlled by *c*. The case of *c* = 0 is equivalent to the *γ* = 2 curve on Fig 3**C**. The performance of the method decreases gradually with the loss of resolution. For A/H3N2 like resolution (*c* = 0.8), the error is close to doubled when compared to the *c* = 0 case. However, for most reassortment rates, the gain in performance over using the naive method remains substantial.

Once MCCs are inferred, we can resolve the subtrees corresponding to an MCC by complementing each others splits. However, this will only result in correct resolutions if the MCCs are correctly identified to begin with and thus requires further validation. Fig 4**C** shows the number of missing splits that are correctly and incorrectly introduced scaled by the total number of missing splits, as a function of *r*^⋆^ (lines with markers) and for both the naive and the *γ* = 2 method. Note, that a split that is resolved in neither tree can not be resolved this way. The maximal resolution, obtained with the knowledge of the true MCCs, is indicated in the figure, showing that for *γ* = 2 the majority of possible resolutions are found while only a small fraction of incorrect splits are introduced.

### Comparison with other methods

To demonstrate the utility of TreeKnit, we compare it to GiRaF [14, 21] and the recently published fully bayesian method CoalRe [9, 17].

GiRaf is, as TreeKnit, based on topological differences between the two trees. Given two trees, it uses the compatibility network of their splits to infer the position of reassortments. However, unlike our method, it does not aim at inferring the whole ARG. Instead, it finds a set of probable reassortments, which may not fully explain the topological differences between segment trees.

CoalRe [9, 17], on the other hand, is a bayesian method that uses a coalescence-reassortment process to construct a probability distribution for ARGs given a sample of sequences. It reconstructs the ARG by sampling from this distribution. It can thus be seen as an extension of usual tree inference methods to the case of genealogies with reassortment. By construction, it uses not only the topology of segment trees, but all the available information contained in the gene sequences.

We compare methods for three different reassortment rates: *r*^⋆^ ∈ {0.01, 0.05, 0.25}. Note that the intermediate value *r*^⋆^ = 0.05 is close to the one that we estimated for segments HA and NA of A/H3N2 influenza. For each value of *r*^⋆^, we simulate five ARGs and extract the segment trees from them. We then simulate the evolution of sequences on these trees, using the JC69 model for simplicity. Mutation rate *μ* is either tuned such that the resulting trees have a close to perfect resolution (*μ*^*high*^), or such that the trees have flu-like polytomies (*μ*^*low*^). We use all three methods to infer reassortment from the simulated sequences. In the case of GiRaF and TreeKnit, trees must first be reconstructed: this is done using the MrBayes program [22] in the case of GiRaF (code provided in the original publication [14]), and using IQ-Tree in the case of TreeKnit [23]. The natural inputs of CoalRe are sequence alignments, and no prior tree reconstruction is needed.

**Fig 5:**
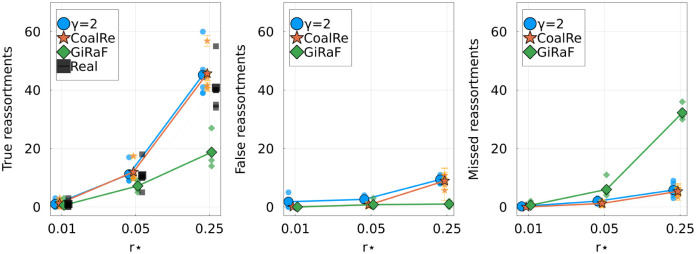
Comparison of TreeKnit with CoalRe [9, 17] and GiRaF [14, 21] on simulated ARGs of 100 leaves. For three reassortment rates, shows the **Left**: number of true reassortments, **Center**: number of false reassortments, and **Right**: number of missed reassortments, for all three methods. Large markers represent the average over 5 simulations for each *r*^⋆^. Smaller markers show results on individual ARGs. Results for each method are slightly shifted on the *r*^⋆^-axis for visibility.

Fig 5 quantifies the accuracy and completeness of the inferences made by the three methods. TreeKnit and CoalRe perform similarly well, with CoalRe reporting slightly less false reassortments. GiRaF misses reassortment events, but very rarely reports a reassortment that did not happen.

TreeKnit has a much shorter runtime than the other methods. For the trees of 100 leaves used here, the average runtime of TreeKnit was 40ms, whereas GiRaF took 40s. Note that these times are small compared what is needed to infer the phylogenetic trees: the IQ-Tree runs took about 30s, while the MrBayes runs took about 20minutes. S11 Fig shows that for our simulated genealogies, the runtime of TreeKnit scales quadratically with the number of leaves. We also observed that GiRaF scales similarly. For very asymmetric trees, which can be the case for influenza genealogies, we observed that the scaling of TreeKnit can become closer to cubic. Finally, CoalRe takes several hours to infer one ARG. TreeKnit thus runs orders of magnitude faster than CoalRe at the cost of a very small reduction accuracy.

### Results on influenza A data

TreeKnit was developed for application to influenza virus data and in particular to seasonal influenza viruses. We have shown (see Fig 3) that TreeKnit accurately infers MCCs of simulated genealogies. But accuracy depends on the reassortment rate and the resolution of the segment trees. In S1 Text, we estimate that A/H3N2 influenza data corresponds to *c* = 0.8 and *r*^⋆^ ≈ 0.05 (S9 and S12(**A**) Figs). S12(**B**) Fig uses simulations to estimate the difficulty of the inference problem in the space of *r* and *c*. Seasonal influenza viruses seem to lie exactly in a region of parameter space where the problem is hard, but most is gained over the naive method. We can thus expect significant improvement in terms of resolution of the segment trees and non-trivial reassortment patterns.

As a mean to investigate the consistency between TreeKnit and other methods, we use TreeKnit on the collection of human influenza H3N2 isolates sequenced and analyzed in [10]. This data has previously been used as a test set by some authors [8, 14]. It consists of about 150 strains sampled in New York between 1999 and 2004.

When considering HA and NA segments, two reassorted clades were found in [10] to be reassortments by manual inspection. The GiRaF method [14] found one more reassorted clade containing a single strain. We applied TreeKnit to this dataset (*γ* = 2), with results shown on S1 and S2 Figs. TreeKnit recovers the two reassortments found in [10] and the additional one found by GiRaF, and also finds three extra reassorted clades: {A/New York/137/2004, A/New York/138/2003}, {A/New York/177/1999}, and a large clade consisting of strains sampled between 2003 and 2004. Manual inspection of the trees suggests that these three clades are indeed reassortments. Note that the fact that we find more reassortments than GiRaF on the same data is consistent with results shown on Fig 5.

## Discussion

Recombination and reassortment are important to reduce mutational load by weeding out deleterious mutations [24] and can facilitate adaptation, especially in rapidly evolving populations [25, 26]. While a number of studies document frequent reassortment in seasonal influenza virus populations [7, 8, 10, 27], reassortment is often ignored because of a lack of methods to infer ARGs at scale. Furthermore, the inferred reassortment history is difficult to visualize and interpret as soon as more than a few reassortment events occurred.

We introduced TreeKnit, a fast and accurate method to infer ancestral reassortment graphs (ARG) from two segment trees. Its underlying idea is to find regions of the genealogy without reassortments, where the two trees have the same topology (MCCs). Intuitively, this is done by “knitting” the trees together starting from the leaves. The method iteratively grows compatible regions and introduces reassortment events until the entire observable ARG is inferred. Strictly speaking the ARG also includes deep reassortment events that are shadowed by more recent dynamics. TreeKnit infers only the observable part of the ARG that is directly connected to the observed taxa. At each stage, the method introduces reassortment events that explain the largest number of incompatibilities and stops when no more reassortment events are found that explain at least *γ* incompatibilities.

The parameter *γ* allows us to tune the behavior of TreeKnit: at *γ* = 1, TreeKnit tries to minimize the number of reassortments, while at *γ* → ∞ only complete subtrees with identical topology are identified. Simulations revealed that *γ* = 2 is a robust choice, giving best results for a wide range of reassortment rates. Note that the method is by design *greedy*, since the function $N_{\gamma }\left(\vec{\sigma }\right)$ only takes into account incompatibilities that are one level above the (effective) leaves. Comparing TreeKnit to CoalRe suggests that the greedy heuristic achieves similar accuracy to more principled bayesian approaches for parameter sets tested, while being orders of magnitude faster.

Efficient inference of ARGs of seasonal influenza viruses should enable deeper insights into the importance of reassortment for antigenic evolution through combination of specific HA and NA variants. So far this has remained unclear, with studies reaching different conclusions [8, 9]. This discordance might in part due to the lack of tools that can infer ARGs of large data sets. In addition, joint analysis of multiple segments should improve resolution of segment trees with polytomies. In parts of the ARG that are common to both segment trees, the sequences of the two segments can be considered jointly, which amounts to roughly doubling the number of mutations per branch. In the future, we plan to extend TreeKnit to multiple and complement it with ARG visualization tools in Nextstrain [28].

## Supporting information

S1 Text Additional analysis, derivations, methods, and robustness checks. (PDF)

S1 Fig Segment trees and tanglegram for the 156 strains studied in [10]. Node support is shown on the sketch in S2 Fig. Strains and branches are colored based on MCCs found by our method (*γ* = 2). MCC 7 does not correspond to a reassortment, as it contains the roots. The list of remaining MCCs is as follows: 1→{A/New York/105/2003}; 2→{A/New York/177/1999}; 3→{A/New York/137/2004, A/New York/138/2003}; 4→{A/New York/52/2004, A/New York/59/2003}; 5→{A/New York/32/2003, A/New York/198/2003, A/New York/199/2003}; 6→{R} (see S2 Fig). Previous studies only found reassortments 1, 4 and 5 [10, 14]. Note that since MCCs have the same topology in the two trees, it is possible to completely disentangle lines of the same color in this plot.
(PNG)

S2 Fig Segment trees for the 156 strains studied in [10]. **Left**: HA segment and **right**: NA segment. Some clades are collapsed for better visibility. Support is indicated for some internal nodes in the form of ultrafast bootstrap values [5], either on the branch above the node or at the right of the node. The 6 MCCs corresponding to a reassortment found by our method (*γ* = 2) are highlighted. One of them involves a clade of 58 strains, shown collapsed and labelled as “R”. The remaining MCC contains all remaining strains as well as the root of both trees, and does not correspond to a reassortment. Previous studies ([10, 14]) only found reassortments for clades {A/New York/52/2004, A/New York/59/2003}, {A/New York/32/2003, A/New York/198/2003, A/New York/199/2003} and {A/New York/105/2003}. We also find clades {A/New York/137/2004, A/New York/138/2003}, {A/New York/177/1999} and {R}.
(PNG)

S3 Fig Illustration of inferrable and non-inferrable reassortments. The ARG is similar to the high-reassortment case of Fig 1 of the main text. Reassortments highlighted by black circles are connected to observed sequences (*i.e*. leaves) by branches shared by the two trees: they will leave traces in the segment trees in the form of topological differences or branch length differences. In the former case, TreeKnit can identify them. On the contrary, reassortments highlighted by black squares are only connected to observed sequences by non-shared branches. Such reassortments leave almost no traces in the segment trees, and TreeKnit does not try to infer them. Note that the coalescence events between different segment lineages that take place below the reassortments marked by squares are also intrisically hard to infer from sequence data.
(PDF)

S4 Fig Evaluation of the robustness of our method with respect to uncertainty in the tree inference. When treeknit is run on two identical trees, there should be exactly one MCC. To test the effect of uncertainty of tree inference on reassortment inference, when we run the tree builder (iqtree) twice on the same alignment. The graph shows the average number of MCCs obtained when applying the algorithm as a function of the bootstrap value below which a branch is collapsed. The average is performed over 10 A/H3N2 HA alignments. Finding more than one MCC implies that some topological differences are introduced by the tree building process, typically because of weakly supported nodes. A minimum bootstrap of 75 is enough to guarantee an MCC inference robust to errors in the inference of the trees.
(PNG)

S5 Fig Equivalent to Fig 3 of the main text for the Yule coalescent. Results of *γ*-methods on simulated ARGs of 100 leaves. Increasing values of *γ* are shown by colored lines, from red to blue.**A**: Number of MCCs found by different methods as a function of the reassortment rate. The real number of MCCs is represented by the marked black line. The naive method (dashed black line) overestimates the number of MCCs for low *ρ*, while the parsimonious one (*γ* = 1) underestimates it for high *ρ*. **B**: True positive rate for reassortments: fraction of inferred reassortments that are indeed present in the real ARG. The low number of reassortments results in a relatively large uncertainty for this quantity for *ρ* ≪ 1. **C**: Distance between inferred and real MCCs for different methods. The distance is based on the variation of information.
(PNG)

S6 Fig Equivalent to Fig 3 of the main text for the Kingman coalescent. Results of *γ*-methods on simulated ARGs of 100 leaves. Increasing values of *γ* are shown by colored lines, from red to blue.**A**: Number of MCCs found by different methods as a function of the reassortment rate. The real number of MCCs is represented by the marked black line. The naive method (dashed black line) overestimates the number of MCCs for low *ρ*, while the parsimonious one (*γ* = 1) underestimates it for high *ρ*. **B**: True positive rate for reassortments: fraction of inferred reassortments that are indeed present in the real ARG. The low number of reassortments results in a relatively large uncertainty for this quantity for *ρ* ≪ 1. **C**: Distance between inferred and real MCCs for different methods. The distance is based on the variation of information.
(PNG)

S7 Fig Equivalent to Fig 3 of the main text for asynchronous samples. Results of *γ*-methods on simulated ARGs of 100 leaves. The backward simulation of the ARGs starts with 25 lineages (leaves), and the 75 remaining leaves are added at a fixed rate over a time ∼2*N*, with *N* the population size. This imitates typical influenza trees with strains coming from different seasons. Increasing values of *γ* are shown by colored lines, from red to blue. **A**: Number of MCCs found by different methods as a function of the reassortment rate. The real number of MCCs is represented by the marked black line. The naive method (dashed black line) overestimates the number of MCCs for low *ρ*, while the parsimonious one (*γ* = 1) underestimates it for high *ρ*. **B**: True positive rate for reassortments: fraction of inferred reassortments that are indeed present in the real ARG. The low number of reassortments results in a relatively large uncertainty for this quantity for *ρ* ≪ 1. **C**: Distance between inferred and real MCCs for different methods. The distance is based on the variation of information.
(PNG)

S8 Fig False predictions for individual branches as a function of *ρ*, for different values of *γ*. Solid lines correspond to branches that are predicted to be shared by the two segment trees, but are not in the real ARG. On the contrary, dashed lines correspond to branches that are predicted to not be shared by the two segment trees, but are shared in the real ARG. The parsimonious method (top-right) makes a lot of the first type of error, and few of the second type. Increasing *γ* transitions between the two types of errors. The naive method (grey dashed line) makes close to only errors of the second type.
(PNG)

S9 Fig Solid lines: Ratio of number of leaves to number of nodes in simulated trees of different sizes, as a function of the amount of parameter *c*, artificially introducing polytomies. Dashed lines: same quantity for A/H3N2 HA trees. For a perfectly resolved tree, this quantity is approximately one half. Polytomies are introduced in simulated trees using the method described in section SA2 of S1 Text. A unique value *c*^⋆^ allows simulated trees of different sizes to reproduce the lack of resolution of influenza trees.
(PNG)

S10 Fig Histogram of the size of polytomies for A/H3N2 HA trees and for simulated ones, with a level of polytomies chosen to reproduce that of influenza. (PNG)

S11 Fig Runtime in seconds as a function of the number of leaves *L*, for different values of *ρ*, in log-log scale. Performed on a single CPU. The linear fit is done for *L* > 10^2^. As expected, runtime is quadratic in the number of leaves of the trees.
(PNG)

S12 Fig **Left**: Estimation of the reassortment rate of segments HA and NA in A/H3N2 influenza. Colored lines show the number of MCCs inferred by *γ*-methods as a function of *γ*. Colors going from blue to red correspond to increasing values of *ρ*. The black line shows the same quantity for the influenza trees. The curve corresponding to influenza lies between the values 0.043 and 0.093. **Right**: VI distance of inferred MCC to the real ones for simulated data and *γ* = 2, as a function of the reassortment rate *ρ* and the amount of polytomies in the trees. The star corresponds to the estimated position of A/H3N2 influenza (HA/NA segments, sequences from the same epidemiological season).
(PNG)

S13 Fig Similar to Fig 5 from the main text, with sequences generated using a lower mutation rate. This results in reconstructed trees with more polytomies. **Left**: number of true reassortments, **Center**: number of false reassortments, and **Right**: number of missed reassortments, for all three methods (Treeknit, GiRaF, CoalRe). Compared to better resolved trees (Fig 5), GiRaF and Coalre infer more false reassortments while Treeknit has more missed reassortments.
(PNG)

S14 Fig Estimation of the convergence of the algorithm for simulated ARGs of *L* leaves: VI distance to real MCCs as a function of the number of iterations of the SA optimization, scaled by *L*. The rhythm of convergence is the same for all curves, indicating that the number of iterations needed to reach convergence should be proportional to *L*.
(PNG)

S15 Fig Estimation of the reproducibility of results for simulated ARGs of *L* leaves: VI distance between two independent runs as function of the number of iterations of the SA optimization, scaled by *L*. For a very low number of iterations, results are close to the naive MCCs, which are the starting point of the optimization. The distance between two runs is maximal for an intermediate number of iterations, and vanishes again as the optimization converges.
(PNG)

S16 Fig PPV for inferred reassortments as a function of their topological distance to the root of one of the segment trees using simulated data. Reassortments inferred close to the root, *i.e*. distance ≲ 4, are more often wrong. A natural explanation for this is that errors made at an early stage of the algorithm will propagate back for the rest of the inference. Additionally, a reassortment close to the leaves can result in a large number of incompatibilities, as it can “move” a clade to a very different part of the tree. Close to the root, there are few lineages left, and thus reassortments have a weaker topological signature.
(PNG)
