# Supplementary material for: TreeKnit: Inferring ancestral reassortment graphs of influenza viruses
Source: PLoS Comput Biol. 2022 Aug 19;18(8):e1010394. doi: 10.1371/journal.pcbi.1010394 (PMC9447925; doi:10.1371/journal.pcbi.1010394)
Supplement: S1 Text — (PDF) [file pcbi.1010394.s001.pdf]

S1 Text

TreeKnit: Inferring Ancestral Reassortment Graphs of influenza viruses

Pierre Barrat-Charlaix, Timothy G. Vaughan, Richard A. Neher,

1 Minimization of N_γ using simulated annealing

Minimizing $N_\gamma(\vec{\sigma})$ amounts to a discrete optimization problem. The functional form of N_γ in Equation (1) allows us to quickly compute a value for a given configuration $\vec{\sigma}$, but does not seem to be amenable to simple optimization. In particular, N_γ can have multiple local & global minima. For this reason, we rely on the general technique of simulated annealing [1].

The algorithm consists having configurations $\vec{\sigma}$ perform a random walk in the energy landscape defined by $N_\gamma(\vec{\sigma})$ at a "temperature" T, using a simple Markov Chain Monte Carlo (MCMC) method. This is equivalent to sampling from the probability distribution $P_T(\vec{\sigma}) \propto e^{-N_\gamma(\vec{\sigma})/T}$. The temperature T is initialized at a high value and slowly brought to 0: this is the cooling process. For an infinitely slow cooling, the sampling process will converge to $\vec{\sigma}^\star$, the minimum of N_γ.

Convergence and reproducibility In practice, cooling cannot be infinitely slow, and there is no guarantee that the algorithm converges to the global minimum, or even that two subsequent runs converge to the same point. However, we observe in our setting that annealing runs give highly reproducible results as long as the cooling speed is inversely proportional to the number of leaves in the trees, that is if the number of iterations of the SA algorithm is proportional to the number of leaves L. Figure S14 shows the distance of inferred MCCs to the real ones as a function of the number of iterations at each temperature T, scaled by the number of leaves L in the ARG. It is clear that the optimization converges for a high enough number of iterations, and that the required number of iterations for proper convergence should be proportional to L.

Reproducibility of results is shown on Figure S15: the distance between MCCs obtained in two independent runs on the same data is plotted against the number of iterations performed at each temperature T, scaled by the number of leaves L. Since the starting point of the optimization is the result of the naive method, the difference between two runs is low for a low number of iterations. It goes through a maximum for an intermediate number, and vanishes again as the optimization converges. The fact that all curves "stack" vertically shows that the number of iterations of the SA algorithm should be chosen proportionally to L.

Runtime The computational complexity is of the minimization can easily be estimated. Computing N_γ for a tree of L leaves results in order L operations. Since we choose the cooling speed of the SA to be inversely proportional to L, we obtain a quadratic complexity $\mathcal{O}(L^2)$. The minimization of N_γ is the most computationally intensive part

of the algorithm for large trees ($L \gtrsim 100$). As a result, we expect the overall runtime to be quadratic in L. This is verified in Figure S11.

2 Simulation of ARGs

We simulate ARGs using a backwards coalescence-reassortment process. The process is initiated with n_0 leaf nodes that all have two segments. Two types of event can then occur:

- Coalescence, with a rate $\nu_c(n)$, where n is the number of remaining lineages in the simulation (n_0 at start). In a coalescence, two nodes are picked at random and their lineages are merged. If these lineages corresponded to different segments or if at least one of the lineages corresponds to two segments, the ancestral node formed will have two segments. Otherwise, it has one. The choice of a functional form for $\nu_c(n)$ is detailed below.

- Reassortment, with a rate $\nu_r = rn_r$ with r constant. n_r is the number of nodes that have two segments. In a reassortment, a node with two segments is chosen at random and its lineage splits backward, giving rise to two ancestors. Each segment goes to one of these ancestors, and these can no longer be reassorted as they only have one segment.

Sampling of events continues until a root for each segment has been found.

The *scaled reassortment rate* is defined using the ratio of reassortment to coalescence rates at the start of the simulation:

$$r^\star = \frac{\nu_r(n_0)}{\nu_c(n_0)}. \quad (1)$$

Importantly, $r^\star$ describes the competition between coalescence and reassortment at the very start of the simulation, when all leaves are present. Consequently, the value $r^\star = 1$ means that the *first event* that will be simulated is equally likely to be a coalescence or a reassortment.

The rate of coalescence can be chosen in several manners. The Kingman coalescent is defined by $\nu_c(n) = n(n-1)/2N$, with timescale N corresponding to a global population size. It is characterized by very short branches close to the tips of the ARG, and long branches that lead back to the root. The Yule coalescent is defined by $\nu_c(n) = (n-1)/N$, and typically has much longer terminal branches than the Kingman's. For results of the main text, we use a custom coalescent model that is designed to reproduce the distribution of tree branch length that is empirically observed in A/H3N2 influenza genealogies. It is defined by $\nu_c = n^{0.2}(n-1)/2N$. All results presented in this article are qualitatively unchanged when using these different types of coalescent models. Figures S5 and S6 are equivalent to Figure 3 from the main text, and show results obtained using the Yule and the Kingman coalescent models.

3 On the reassortment rate

We explain here the choice of the definition of $r^\star$ in Equation 2, and the difference with the usual definition of the reassortment rate ρ in literature. In the literature, the reassortment rate ρ is usually defined as rT_2, where T_2 is the pairwise coalescence time (with our notation, we have $T_2 = \nu_c(2)^{-1}$). In other words, ρ is the average number of reassortments occurring on a lineage during the time it takes for a pair of leaves to

coalesce to a common ancestor. If the value of T_2 is known in years, as in the case for the influenza virus, then ρ is measured in reassortments per lineage per year.

However, using ρ as a control parameter for the strength of reassortment in simulations is impractical: at a fixed ρ, the properties of the MCCs will vary with n and with ν_c. As an example, consider an ARG generated using a Kingman coalescent: $\nu_c(n) = n(n-1)/N$. Adding more leaves to the simulation will result in an initial coalescence rate that is much higher than the reassortment rate, and many coalescences will take place before the first reassortment. As a result, going from a low n to a large n will take us from a situation like the one in the right panel of Figure 1 of the main text (many reassortments, small MCCs, very different segment trees) to one like the left panel of the same figure (very few reassortments, large MCCs, very similar segment trees). ρ alone is therefore not a good indicator of the different regimes shown in Figure 1.

For this reason, we construct another reassortment rate $r^\star$, defined in Equation 1, which controls the relative strength of coalescence and reassortment. $r^\star$ is indicative of the different regimes depicted in Figure 1: the transitions between low, intermediate and high levels of reassortments will take place in the region $r^\star \simeq 10^{-2} - 10^{-1}$, irrespective of the number of leaves and of the coalescent model used. In particular, the choice of $r^\star$ as a scale for the reassortment rate allows Figures 3, S5 and S6 to use the same range of values on the x-axis, even though they correspond to three different coalescent models.

ρ and $r^\star$ are related in a straightforward way. First, we write the coalescent rate as follows:

$$\nu_c(n) = \left(\frac{n}{2}\right)^\alpha \frac{n-1}{T_2},$$

with the Kingman coalescent corresponding to $\alpha = 1$, the Yule coalescent to $\alpha = 0$, and our custom coalescent model to $\alpha = 0.2$ (up to a proportionality constant). Note that T_2 defines the pairwise coalescence time $T_2 = \nu_c(2)$. From there, we immediately obtain

$$\rho = \left(\frac{n}{2}\right)^\alpha r^\star. \tag{2}$$

In Figure S12**A**, we estimate $r^\star \simeq 0.06$ in the case of segments HA and NA of A/H3N2 influenza. Since this was estimated using a coalescent with $\alpha = 0.2$ and with $n = 100$ leaves, we have for the reassortment rate $\rho \simeq 0.13$ reassortments per lineage per year. This is smaller but in the same order of magnitude than estimations obtained by the CoalRe method [2].

4 Introducing polytomies in simulated trees

To investigate the case of incompletely resolved trees, we need to introduce polytomies in the trees that come from the simulated ARG. We do so in the following way: for each each branch of length t in a given tree, we remove it with an exponentially decreasing probability $P_r = \exp(-t/cN)$. Here, c is a parameter that can be varied between 0 (no branches removed, binary trees) and ∞ (star trees, completely unresolved), and N is the population size that was used in the coalescent model. If sequences were simulated on the trees by a simple evolutionary process, this would amount to remove branches with no mutations, with cN acting as the inverse of a mutation rate.

Figure S9 shows the ratio of the number of leaves to the number of internal nodes for A/H3N2 HA trees (dashed lines) as well as for simulated ones with varying values of c (solid lines). This ratio should be one half in the case of perfectly resolved trees, which are then binary, and can go up to one for fully unresolved trees (star-tree). As expected, the ratio increases with the number of strains used to build the trees. The figure shows that a unique value $c^\star$ allows us to replicate the ratio of leaves to internal nodes of HA trees for different values of the number of leaves.

Note that this result is a consequence of the specific coalescent-reassortment model that we used, discussed in section SA 2. In particular, a different choice of the coalescence rate would make $c^\star$ depend on the number of leaves. In addition, Figure S10 shows that the simulated trees can closely reproduce the distribution of polytomy sizes. Together, these results show that we can reliably replicate the lack of resolution of influenza gene trees in our simulations, and therefore estimate its effect on the reconstruction of the ARG.

5 Choosing among equivalent topological solutions using a likelihood test

Our algorithm, schematized in main text Figure 2, consists in optimizing the discrete function N_γ as a function of the set $\vec{\sigma}$ of effective leaves removed or kept in the tree. The optimal configuration $\vec{\sigma}^\star$ gives us the leaves to remove, that is the found MCCs for this iteration of the algorithm, and the leaves to keep. For certain input trees or reduced trees, it is possible that N_γ has several minima. These corresponds to sets of putative MCCs that are equivalently good solutions in terms of topology only. A very simple case of such a situation is for the set of the two following trees (written as Newick strings, without branch length for simplicity): `((A,B),C)` and `(A,(B,C))`. In this case, three incompatibilities exist when no leaf is removed, and removing any of the leaves `A`, `B` or `C` fixes all incompatibilities. Therefore, for $\gamma < 3$, three topologically equivalent solutions are found by the algorithm.

In such cases, we need to use information beyond topology to break the degeneracy of solutions. The most intuitive information at our disposal is of course branch length. In order to use branch lengths to pick one solution, we derive here a simple likelihood test. First, we make some simple hypothesis over the evolutionary process that gave rise to the tree and over the signification of branch lengths. For each segment i, we call L_i the length of its sequence, μ_i its per site mutation rate, $\{t_i\}$ the length of branches of its tree. The branch length t_i is interpreted as the number of mutations *per sequence site* that occurred on said branch.

For a given branch in the tree of segment i, we call T_i the physical time corresponding to this branch. By definition of the mutation rate, the expected length of this branch in the tree is therefore $\langle t_i \rangle = \mu_i T_i$. However, the actual observed value of t_i may not be equal to the expected one because of the stochastic nature of mutation events. We assume here that the probability of observing a given number of mutations $n_i = L_i t_i$ on this branch is given by a Poisson distribution with mean $n_i^\star = L_i \mu_i T_i$:

$$P(t_i|T_i) = P(n_i|n_i^\star) = \frac{e^{-n_i^\star} n_i^{\star\, n_i^\star}}{n_i!}. \quad (3)$$

We now make two observations about our algorithm. The first is that removing a leaf from a configuration $\vec{\sigma}$ amounts to making that leaf an MCC and introducing a reassortment above it. Therefore, the associated prediction is that the branches joining said leaf to its ancestors in the two segment trees are not shared in the ARG and can be of two different lengths and correspond to two different physical times. We can attribute a probability of observing branch lengths t_1 and t_2 above said leaf given that we predict these branches are not shared:

$$P^{ns}(t_1, t_2; T_1, T_2) = P(t_1|T_1) \cdot P(t_2|T_2),$$

where T_1 and T_2 are the physical times corresponding to the two branches. The most likely values of T_1 and T_2 are those that respectively maximize $P(t_1|T_1)$ and $P(t_2|T_2)$,

giving $T_1^\star = t_1/\mu_1$ and $T_2^\star = t_2/\mu_2$. We therefore define the likelihood of the "not shared branches" prediction as

$$\mathcal{L}^{ns}(t1, t2) = P(t_1|T_1^\star) \cdot P(t_2|T_2^\star). \tag{4}$$

The second observation is that if there is no incompatibility for a given leaf, the prediction of the algorithm is that no reassortment occurred above it. Therefore, the branches above the leaf will be shared in the ARG, and must correspond to the same physical time T. Proceeding as above, we compute the probability of observing branch length t_1 and t_2 if predicting that they are shared branches:

$$P^s(t_1, t_2; T) = P(t_1|T) \cdot P(t_2|T).$$

It can easily be shown that if the mutation rates are similar, the most likely value of T, that is the one maximizing $P^s(t_1, t_2; T)$, is

$$T^\star = \frac{L_1 t_1 + L_2 t_2}{L_1 + L_2}. \tag{5}$$

In turn, this allows us to define a likelihood for the "shared branches" prediction:

$$\mathcal{L}^s(t1, t2) = P(t_1|T^\star) \cdot P(t_2|T^\star). \tag{6}$$

For a given configuration $\vec{\sigma}$, we now proceed as follows to score it using branch lengths. For each leaf n such that $\sigma_n = 0$, we count a likelihood ratio score of $L^{ns}(t_1, t_2)/L^s(t_1, t_2)$, where t_1 and t_2 are the branch lengths above n in each segment tree. Inversely, for each leaf n such that $\sigma_n = 1$ and that does not have any incompatibility above it, we count a likelihood ratio score of $L^s(t_1, t_2)/L^{ns}(t_1, t_2)$. These scores are multiplied, giving us an overall score for $\vec{\sigma}$. When several optimal configurations are found after optimization, the one with the highest likelihood ratio score is chosen.

Even though this likelihood test is necessary to the completeness of our method, we find in practice that it has little influence over the results. The quality of the inferred MCCs is only marginally improved when using it. This likely comes from the relatively infrequent occurrence of conflicting degenerate minima of N_γ.

6 Simulating sequences

For comparison with CoalRe. Nucleotide sequences of length 1000 are simulated independently on each segment tree, using a JC69 model.

7 Robustness with regard to tree inference

The method described in this article relies entirely on topological differences between trees. This means that topological mistakes done in the reconstruction of the trees from sequences can have an important impact on the results. In particular, branches that are not well supported will tend to introduce many spurious incompatibilities between trees.

To mitigate this effect, we pre-process trees obtained from A/H3N2 influenza sequences in the following way:

- If sequences are of length L, every branch shorter than $1/(2L)$ are removed. This strongly reduces the number of branches on which a mutation is unlikely.
- Branches with a bootstrap support smaller than $b_0 = 75$ are removed (ultra fast bootstrap test in iqtree [3]).

To determine b_0, we used the following test. For a given alignment, we infer two trees independently, keeping only branches with bootstrap support higher than b. We then give these two trees as an input to our method and infer a set of MCCs. In principle, the two trees should be exactly the same, and we should therefore find one MCC containing all the leaves. Figure S4 plots the average number of MCCs found in this way as a function of b. It is clear that for $b > 75$, no spurious topological incompatibility remain.

References

[1] Kirkpatrick S, Gelatt CD, Vecchi MP. Optimization by Simulated Annealing. Science. 1983;220(4598):671–680. doi:10.1126/science.220.4598.671.

[2] Müller NF, Stolz U, Dudas G, Stadler T, Vaughan TG. Bayesian inference of reassortment networks reveals fitness benefits of reassortment in human influenza viruses. Proceedings of the National Academy of Sciences. 2020;117(29):17104–17111. doi:10.1073/pnas.1918304117.

[3] Minh BQ, Schmidt HA, Chernomor O, Schrempf D, Woodhams MD, von Haeseler A, et al. IQ-TREE 2: New Models and Efficient Methods for Phylogenetic Inference in the Genomic Era. Molecular Biology and Evolution. 2020;doi:10.1093/molbev/msaa015.
