# Supplementary figures and images for: TreeKnit: Inferring ancestral reassortment graphs of influenza viruses
Source: PLoS Comput Biol. 2022 Aug 19;18(8):e1010394. doi: 10.1371/journal.pcbi.1010394 (PMC9447925; doi:10.1371/journal.pcbi.1010394)

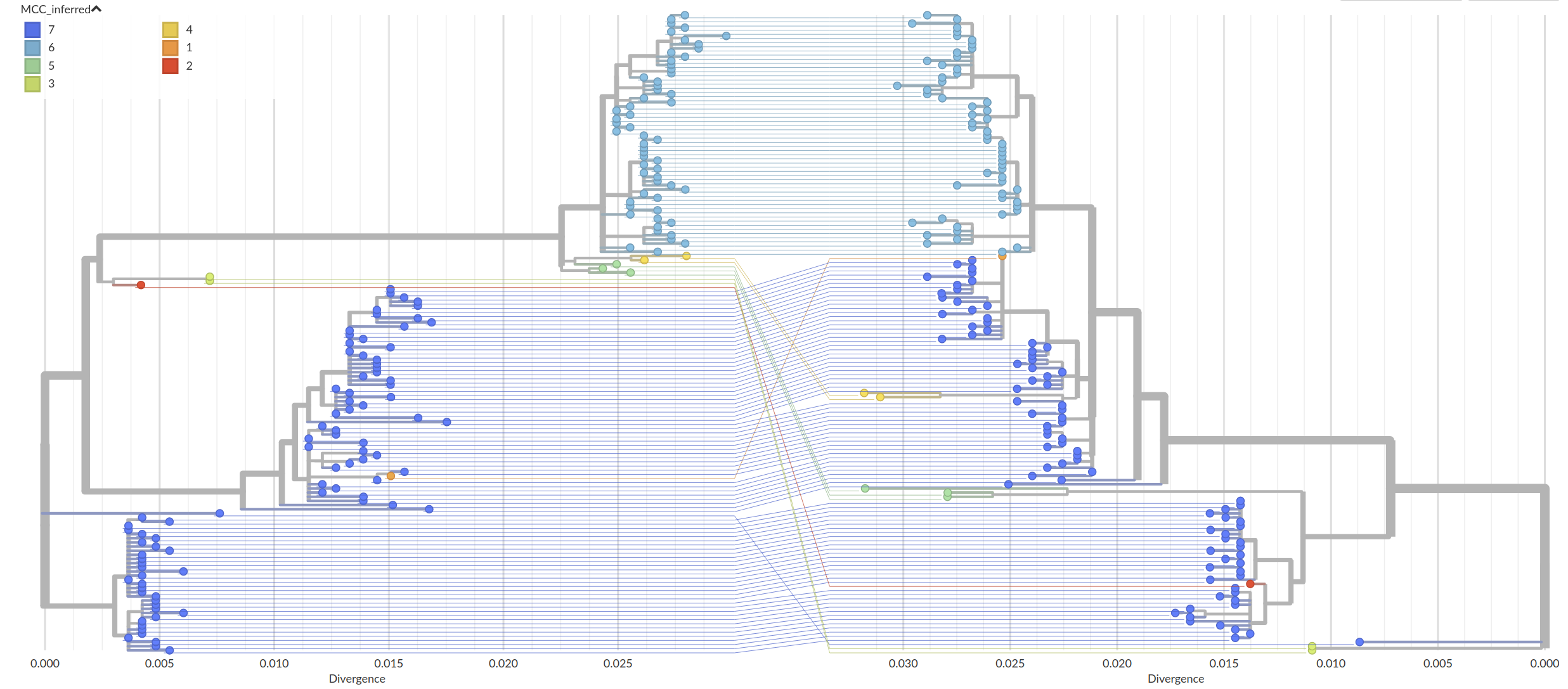

Supplement: S1 Fig — Node support is shown on the sketch in S2 Fig. Strains and branches are colored based on MCCs found by our method (γ = 2). MCC 7 does not correspond to a reassortment, as it contains the roots. The list of remaining MCCs is as follows: 1→{A/New York/105/2003}; 2→{A/New York/177/1999}; 3→{A/New York/137/2004, A/New York/138/2003}; 4→{A/New York/52/2004, A/New York/59/2003}; 5→{A/New York/32/2003, A/New York/198/2003, A/New York/199/2003}; 6→{R} (see S2 Fig). Previous studies only found reassortments 1, 4 and 5 [10, 14]. Note that since MCCs have the same topology in the two trees, it is possible to completely disentangle lines of the same color in this plot. (PNG) [file pcbi.1010394.s002.png]

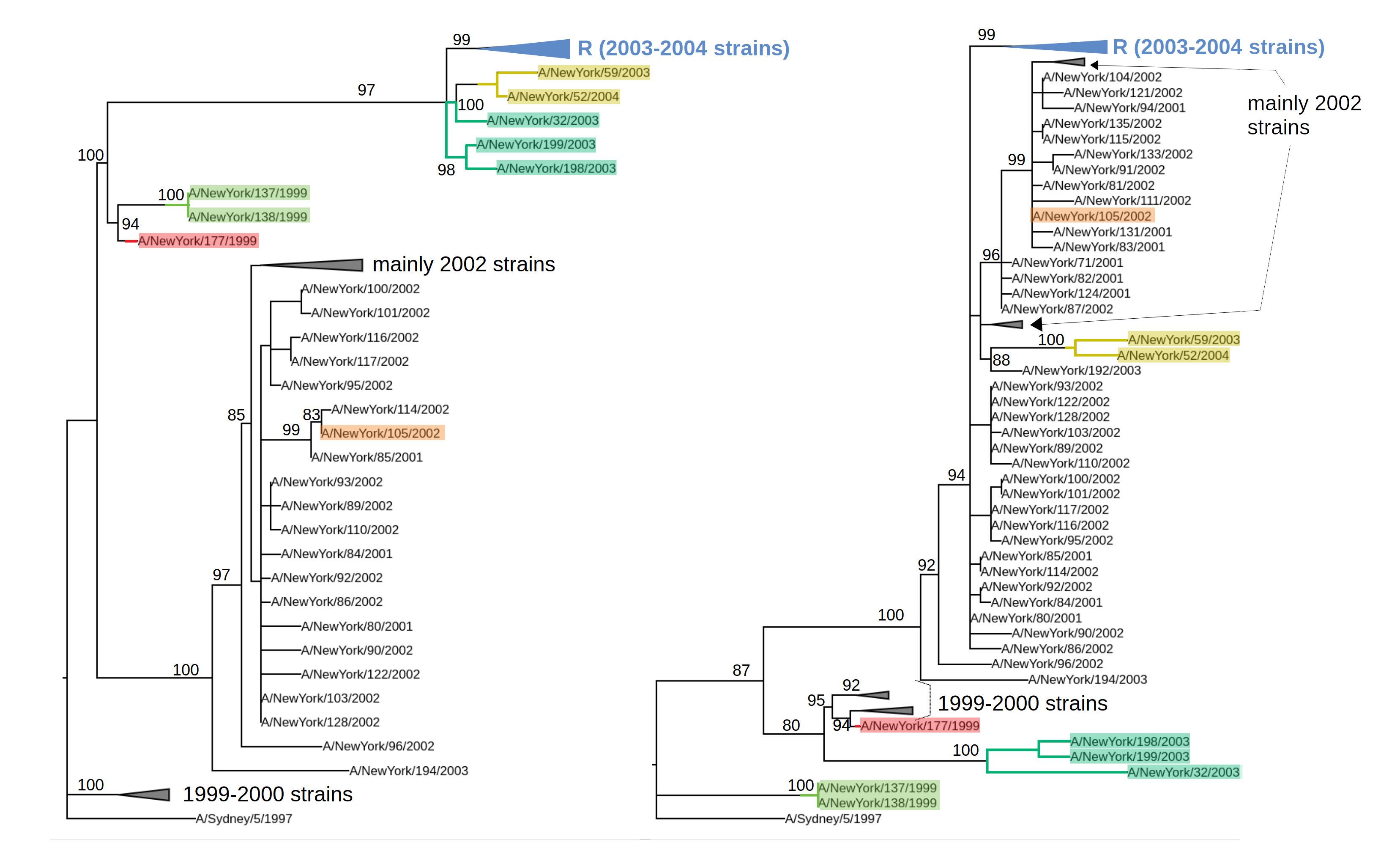

Supplement: S2 Fig — Left: HA segment and right: NA segment. Some clades are collapsed for better visibility. Support is indicated for some internal nodes in the form of ultrafast bootstrap values [5], either on the branch above the node or at the right of the node. The 6 MCCs corresponding to a reassortment found by our method (γ = 2) are highlighted. One of them involves a clade of 58 strains, shown collapsed and labelled as “R”. The remaining MCC contains all remaining strains as well as the root of both trees, and does not correspond to a reassortment. Previous studies ([10, 14]) only found reassortments for clades {A/New York/52/2004, A/New York/59/2003}, {A/New York/32/2003, A/New York/198/2003, A/New York/199/2003} and {A/New York/105/2003}. We also find clades {A/New York/137/2004, A/New York/138/2003}, {A/New York/177/1999} and {R}. (PNG) [file pcbi.1010394.s003.png]

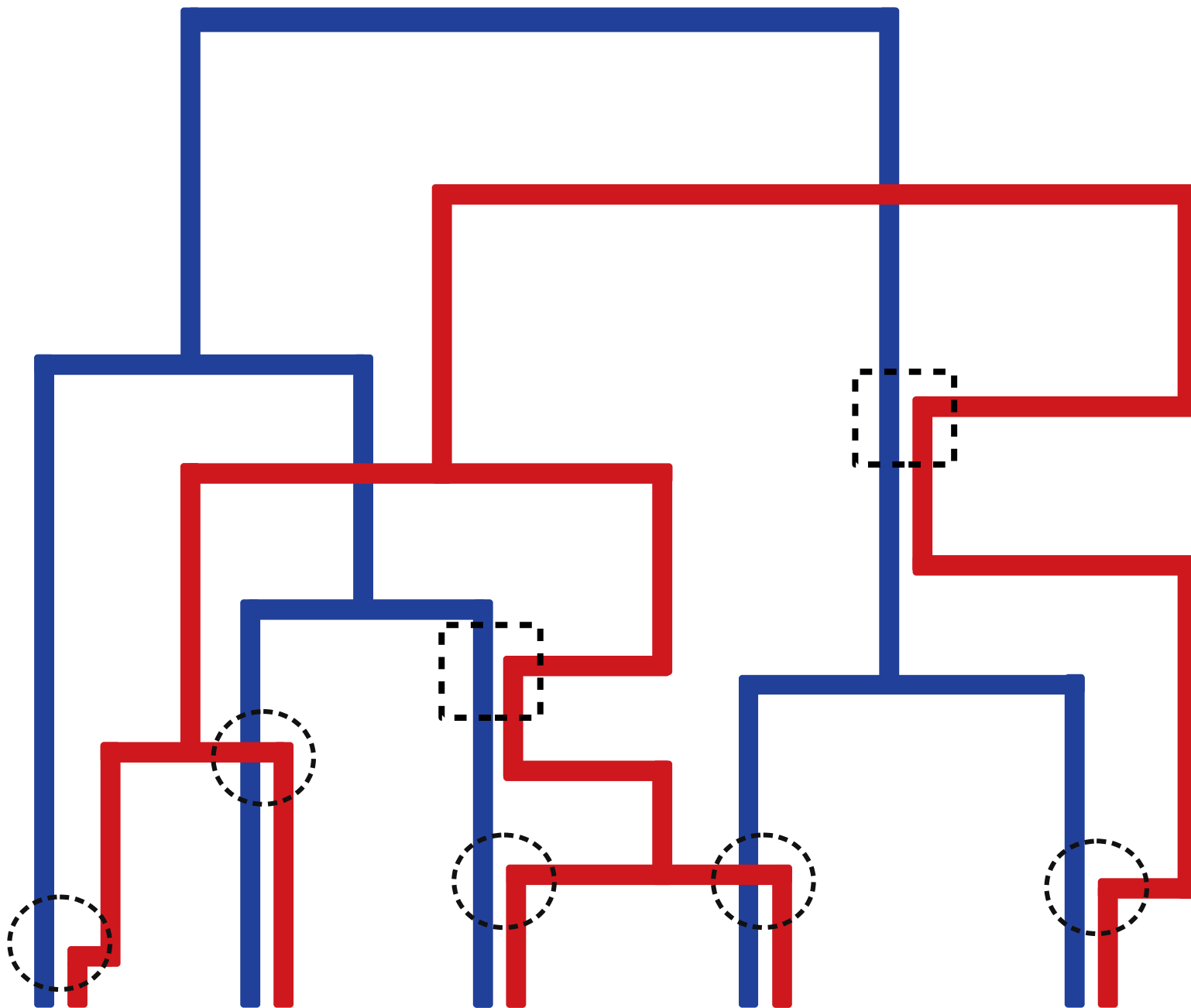

Supplement: S3 Fig — The ARG is similar to the high-reassortment case of Fig 1 of the main text. Reassortments highlighted by black circles are connected to observed sequences (i.e. leaves) by branches shared by the two trees: they will leave traces in the segment trees in the form of topological differences or branch length differences. In the former case, TreeKnit can identify them. On the contrary, reassortments highlighted by black squares are only connected to observed sequences by non-shared branches. Such reassortments leave almost no traces in the segment trees, and TreeKnit does not try to infer them. Note that the coalescence events between different segment lineages that take place below the reassortments marked by squares are also intrisically hard to infer from sequence data. (PDF) [file pcbi.1010394.s004.pdf]

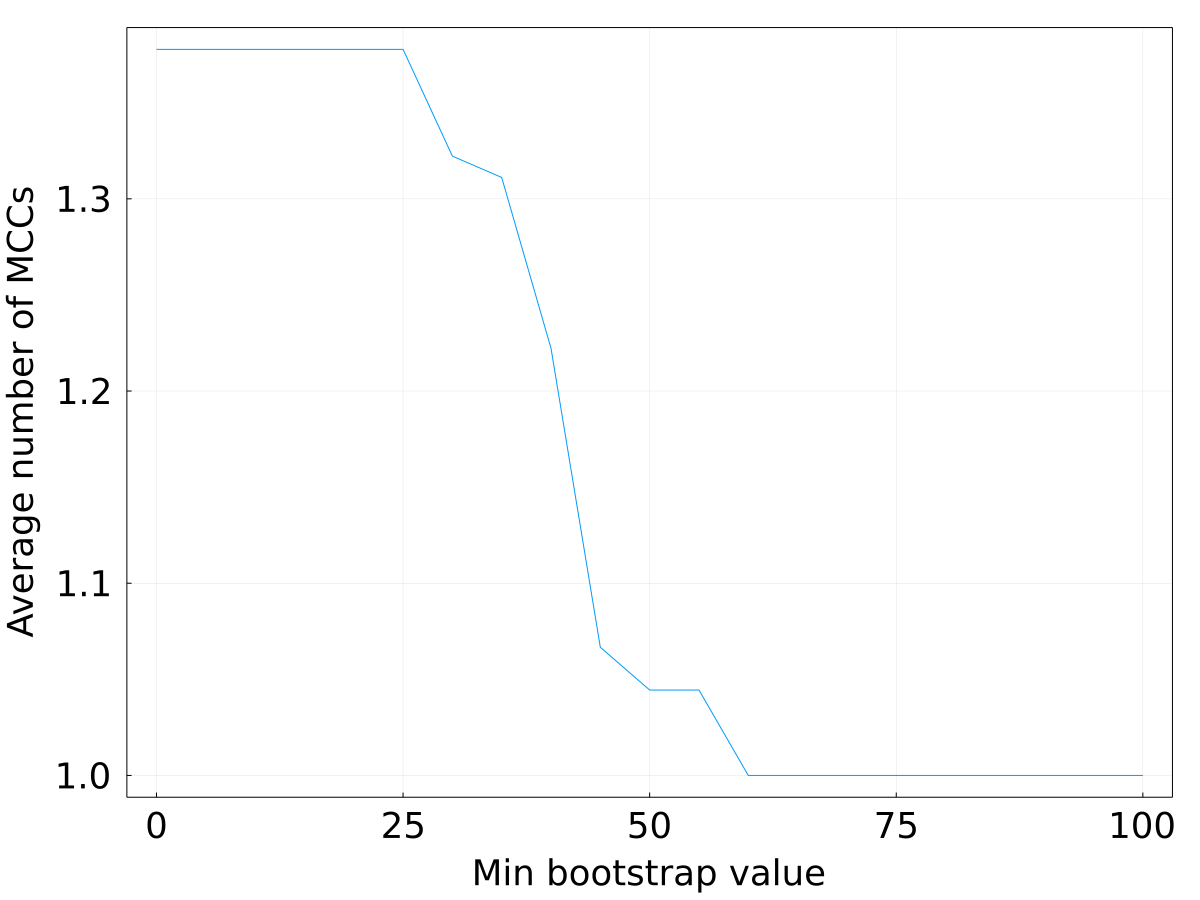

Supplement: S4 Fig — When treeknit is run on two identical trees, there should be exactly one MCC. To test the effect of uncertainty of tree inference on reassortment inference, when we run the tree builder (iqtree) twice on the same alignment. The graph shows the average number of MCCs obtained when applying the algorithm as a function of the bootstrap value below which a branch is collapsed. The average is performed over 10 A/H3N2 HA alignments. Finding more than one MCC implies that some topological differences are introduced by the tree building process, typically because of weakly supported nodes. A minimum bootstrap of 75 is enough to guarantee an MCC inference robust to errors in the inference of the trees. (PNG) [file pcbi.1010394.s005.png]

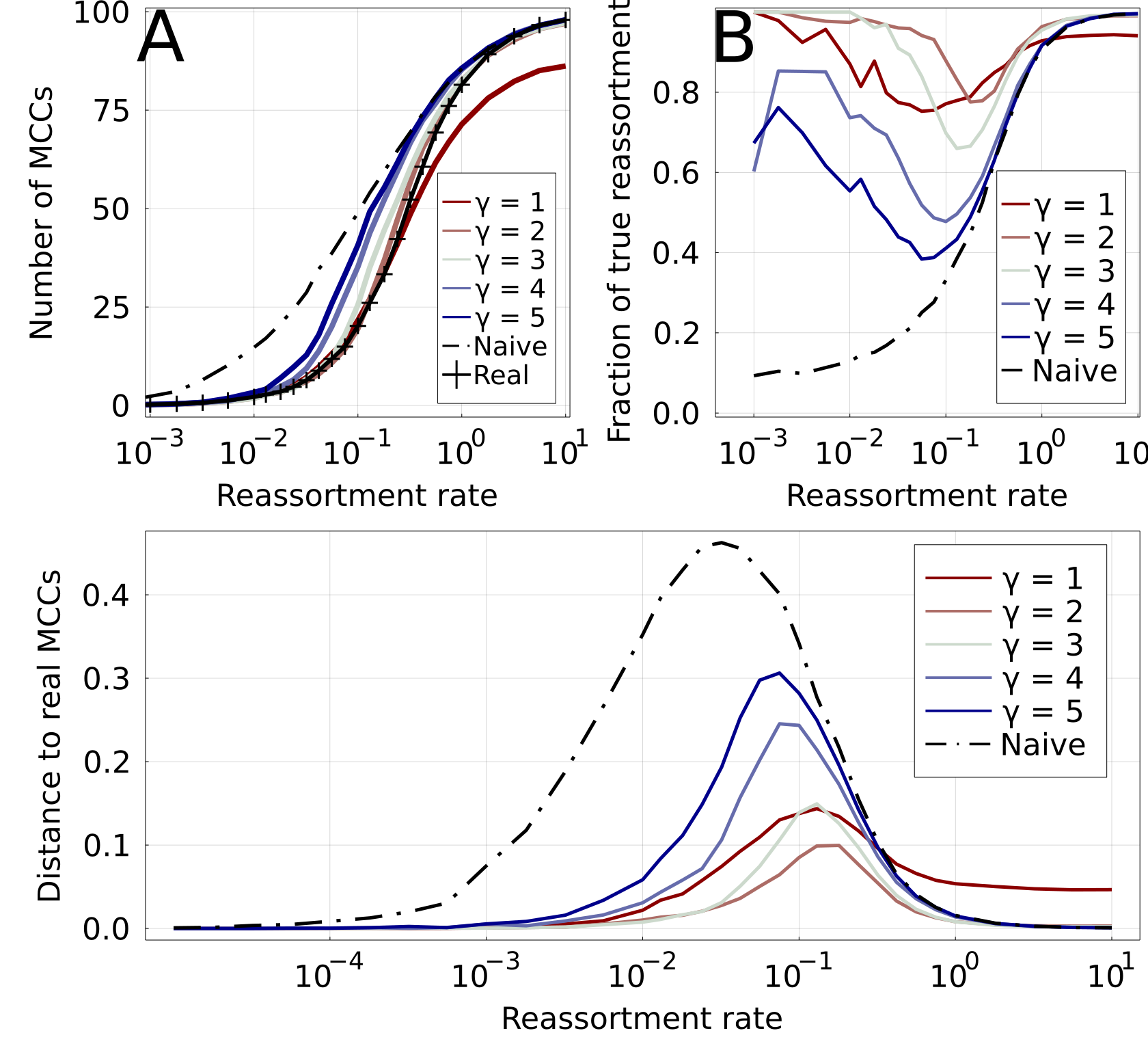

Supplement: S5 Fig — Results of γ-methods on simulated ARGs of 100 leaves. Increasing values of γ are shown by colored lines, from red to blue.A: Number of MCCs found by different methods as a function of the reassortment rate. The real number of MCCs is represented by the marked black line. The naive method (dashed black line) overestimates the number of MCCs for low ρ, while the parsimonious one (γ = 1) underestimates it for high ρ. B: True positive rate for reassortments: fraction of inferred reassortments that are indeed present in the real ARG. The low number of reassortments results in a relatively large uncertainty for this quantity for ρ ≪ 1. C: Distance between inferred and real MCCs for different methods. The distance is based on the variation of information. (PNG) [file pcbi.1010394.s006.png]

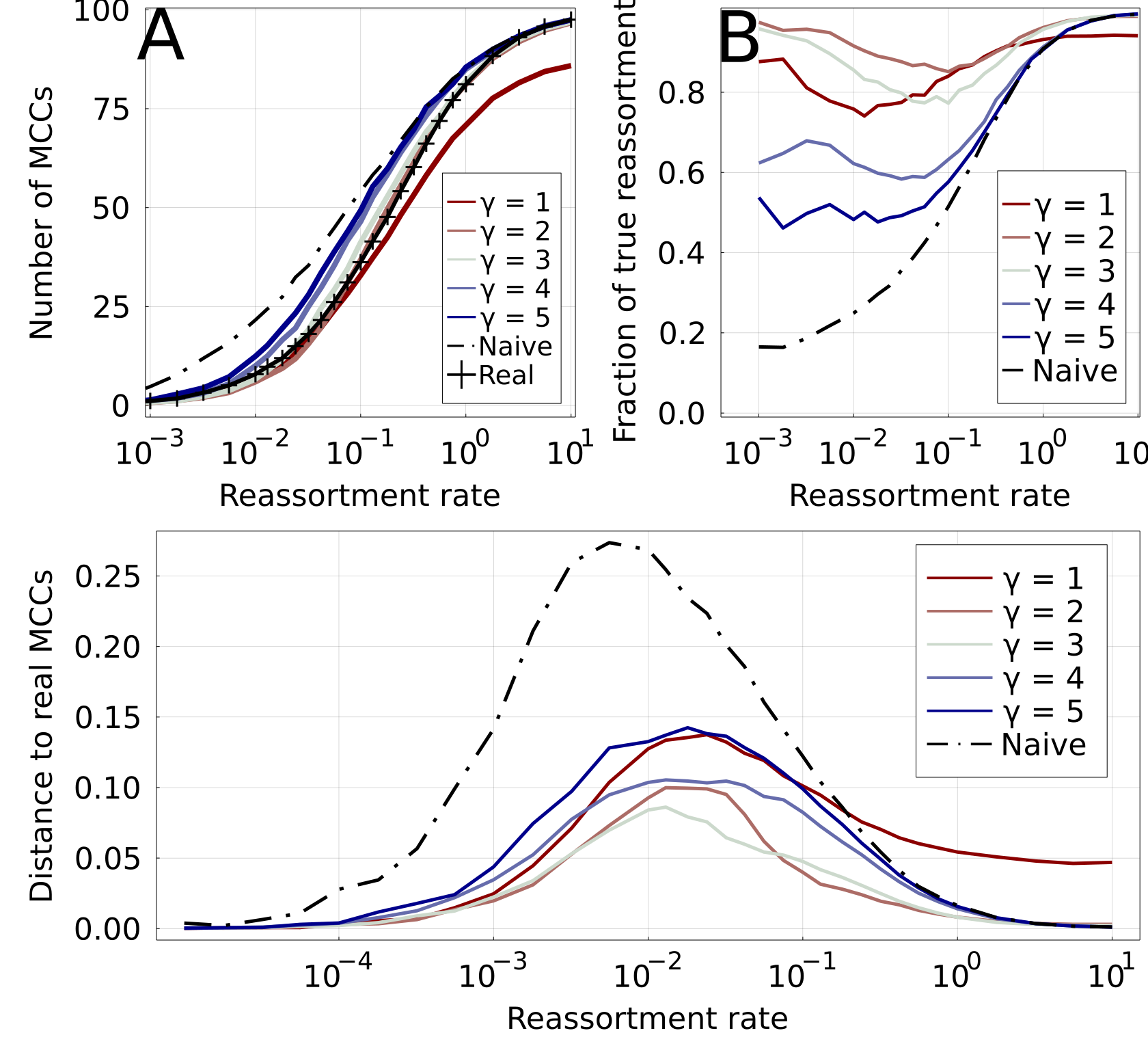

Supplement: S6 Fig — Results of γ-methods on simulated ARGs of 100 leaves. Increasing values of γ are shown by colored lines, from red to blue.A: Number of MCCs found by different methods as a function of the reassortment rate. The real number of MCCs is represented by the marked black line. The naive method (dashed black line) overestimates the number of MCCs for low ρ, while the parsimonious one (γ = 1) underestimates it for high ρ. B: True positive rate for reassortments: fraction of inferred reassortments that are indeed present in the real ARG. The low number of reassortments results in a relatively large uncertainty for this quantity for ρ ≪ 1. C: Distance between inferred and real MCCs for different methods. The distance is based on the variation of information. (PNG) [file pcbi.1010394.s007.png]

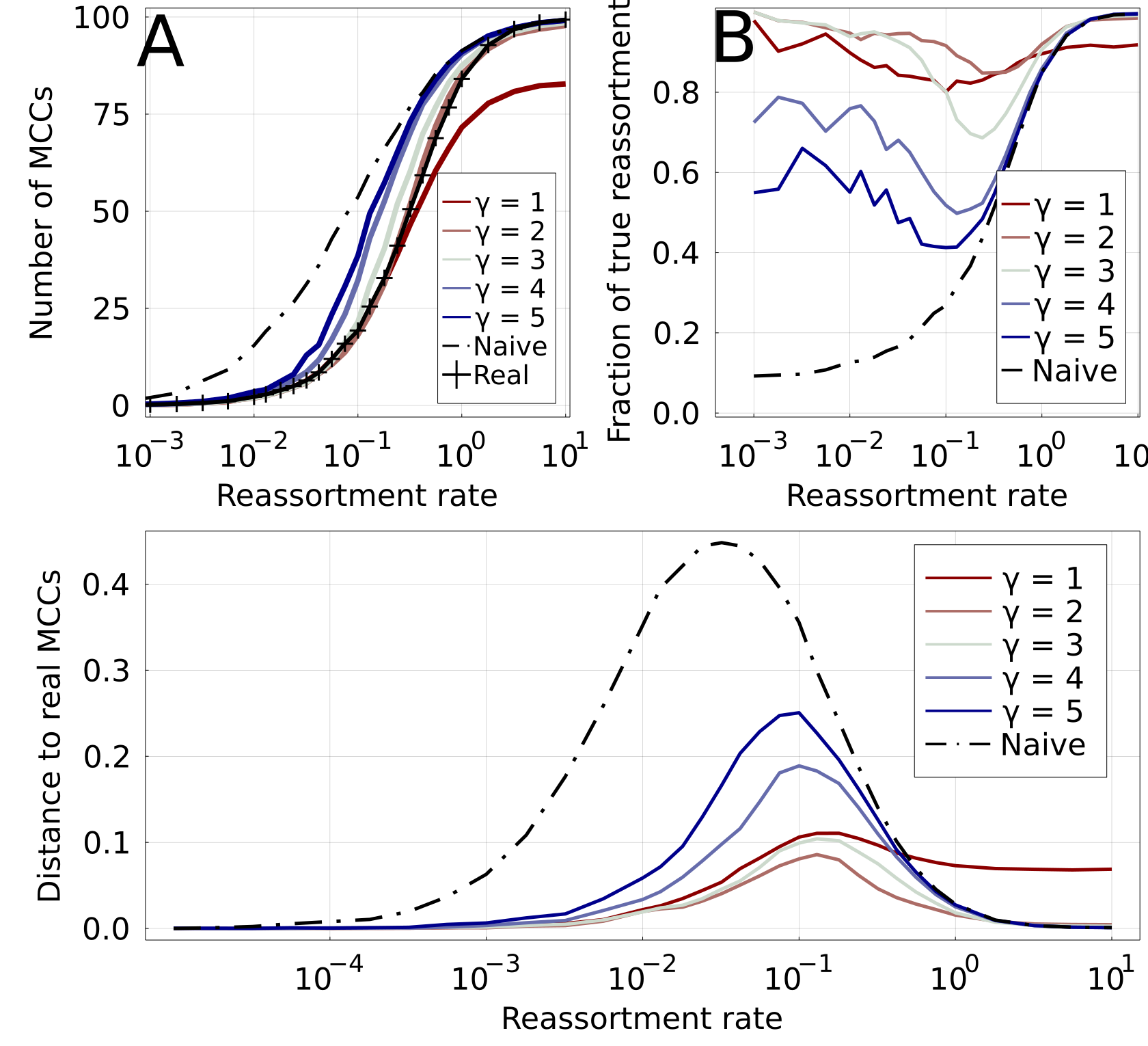

Supplement: S7 Fig — Results of γ-methods on simulated ARGs of 100 leaves. The backward simulation of the ARGs starts with 25 lineages (leaves), and the 75 remaining leaves are added at a fixed rate over a time ∼2N, with N the population size. This imitates typical influenza trees with strains coming from different seasons. Increasing values of γ are shown by colored lines, from red to blue. A: Number of MCCs found by different methods as a function of the reassortment rate. The real number of MCCs is represented by the marked black line. The naive method (dashed black line) overestimates the number of MCCs for low ρ, while the parsimonious one (γ = 1) underestimates it for high ρ. B: True positive rate for reassortments: fraction of inferred reassortments that are indeed present in the real ARG. The low number of reassortments results in a relatively large uncertainty for this quantity for ρ ≪ 1. C: Distance between inferred and real MCCs for different methods. The distance is based on the variation of information. (PNG) [file pcbi.1010394.s008.png]

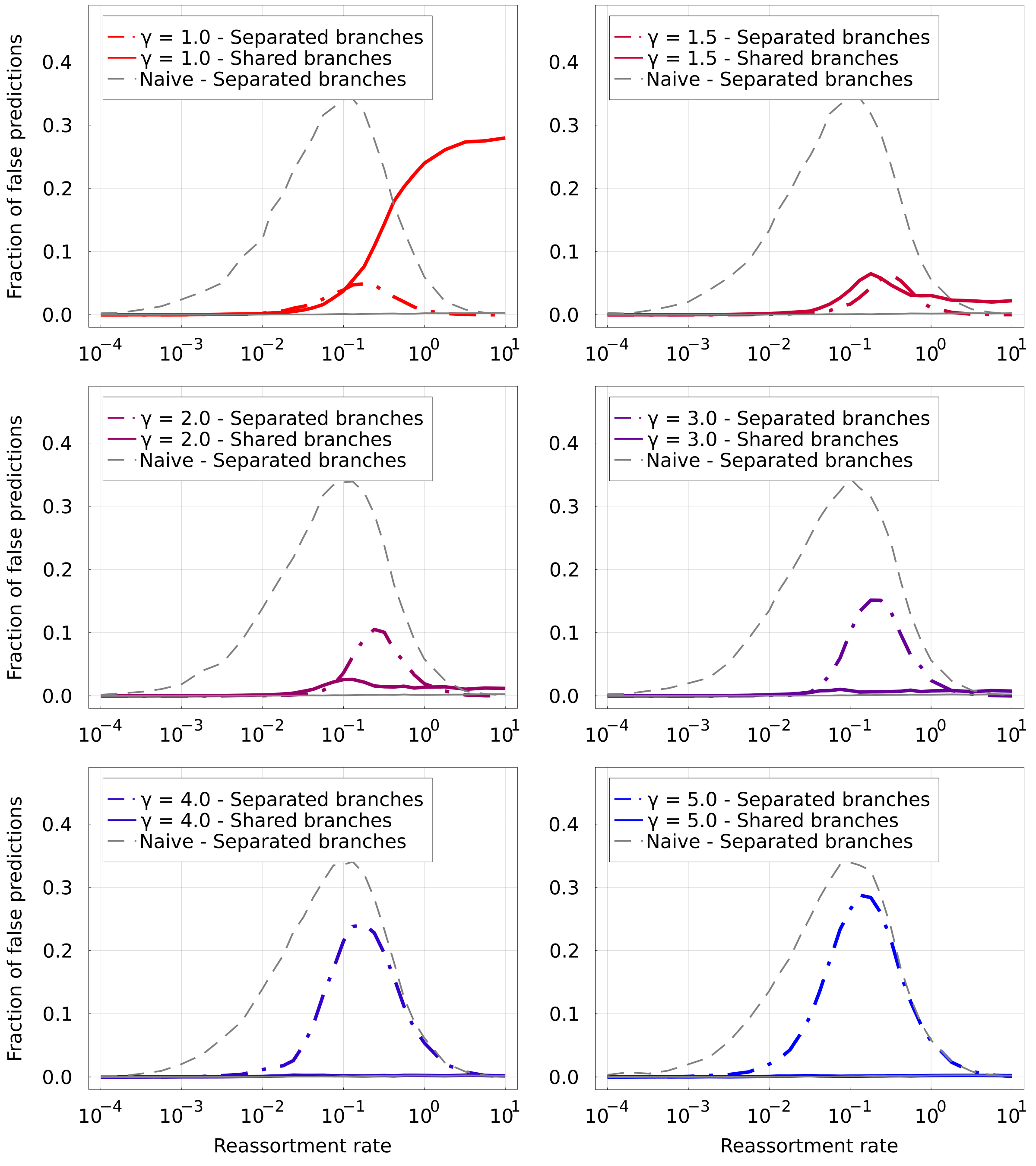

Supplement: S8 Fig — Solid lines correspond to branches that are predicted to be shared by the two segment trees, but are not in the real ARG. On the contrary, dashed lines correspond to branches that are predicted to not be shared by the two segment trees, but are shared in the real ARG. The parsimonious method (top-right) makes a lot of the first type of error, and few of the second type. Increasing γ transitions between the two types of errors. The naive method (grey dashed line) makes close to only errors of the second type. (PNG) [file pcbi.1010394.s009.png]

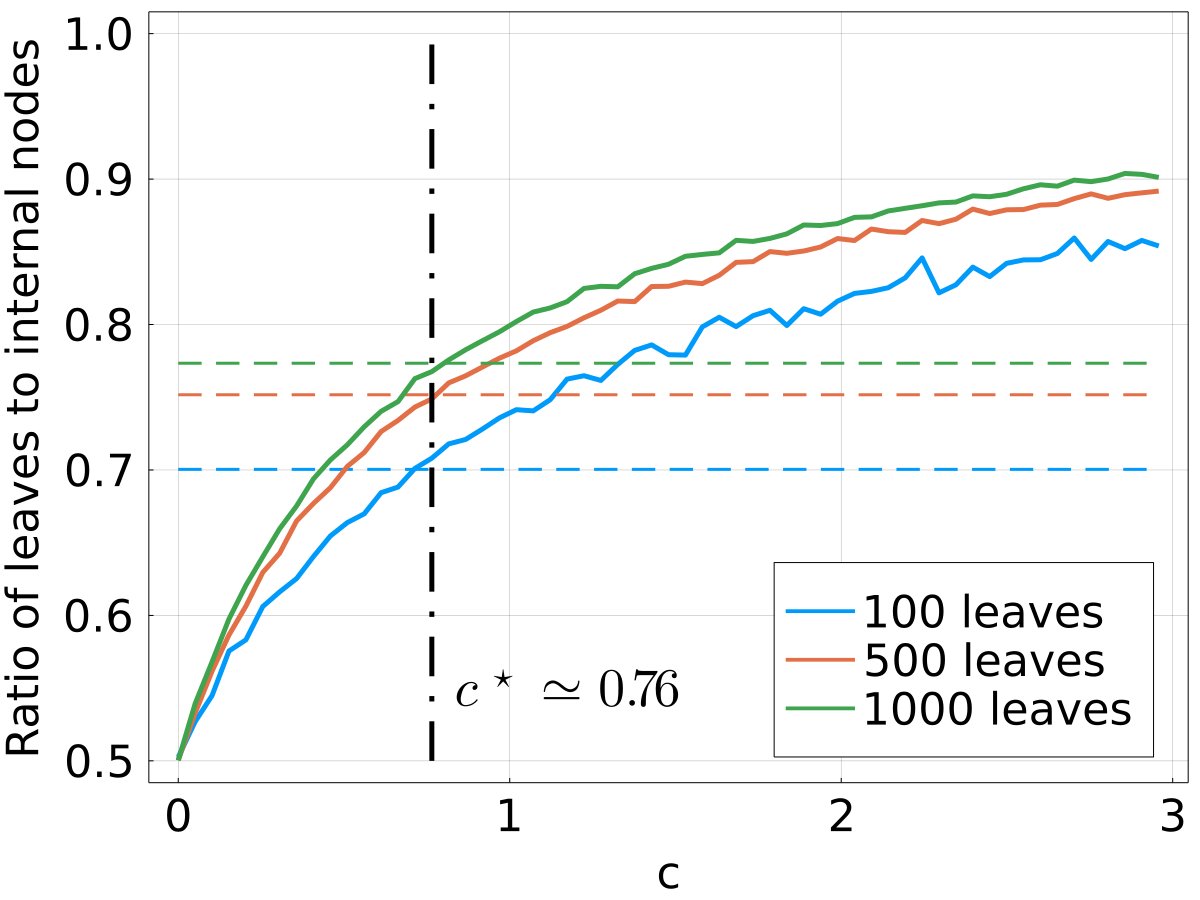

Supplement: S9 Fig — Dashed lines: same quantity for A/H3N2 HA trees. For a perfectly resolved tree, this quantity is approximately one half. Polytomies are introduced in simulated trees using the method described in section SA2 of S1 Text. A unique value c⋆ allows simulated trees of different sizes to reproduce the lack of resolution of influenza trees. (PNG) [file pcbi.1010394.s010.png]

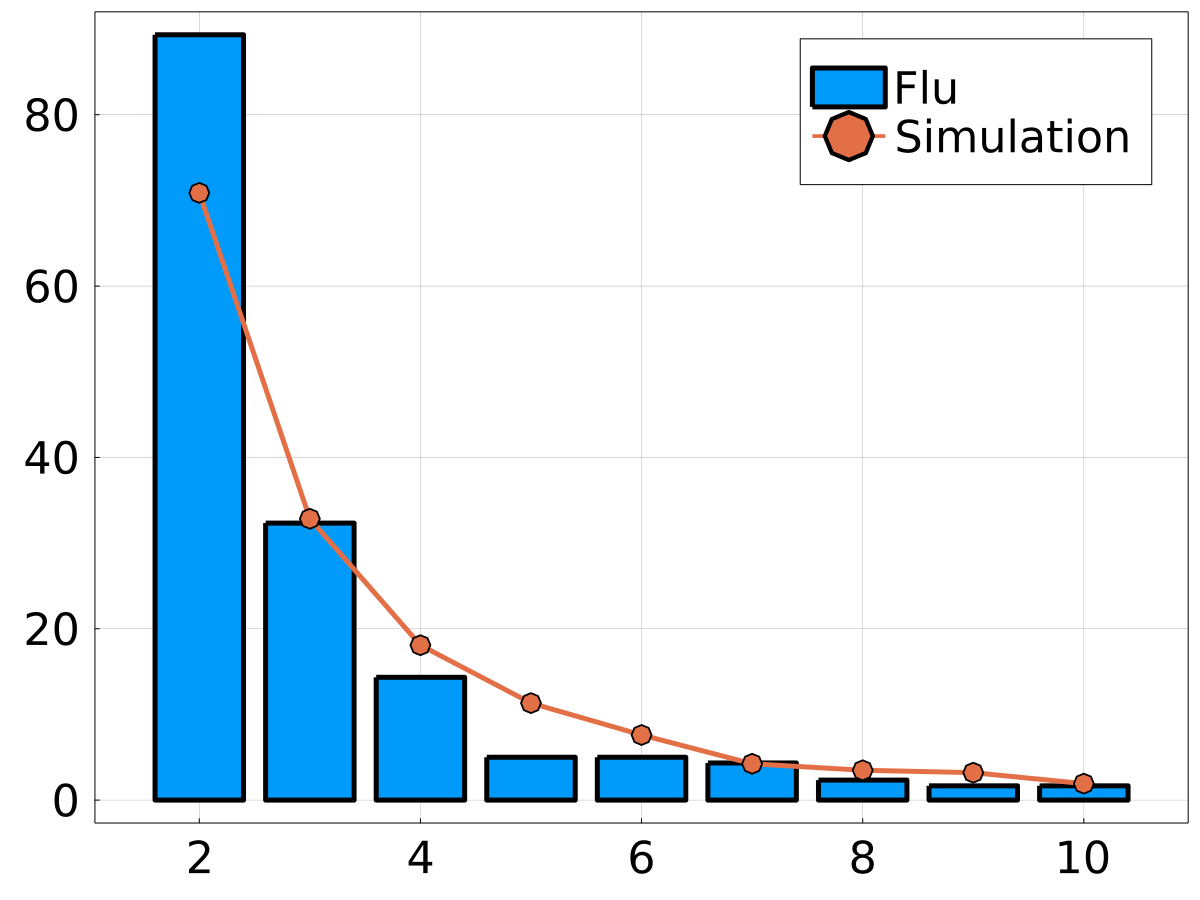

Supplement: S10 Fig — (PNG) [file pcbi.1010394.s011.png]

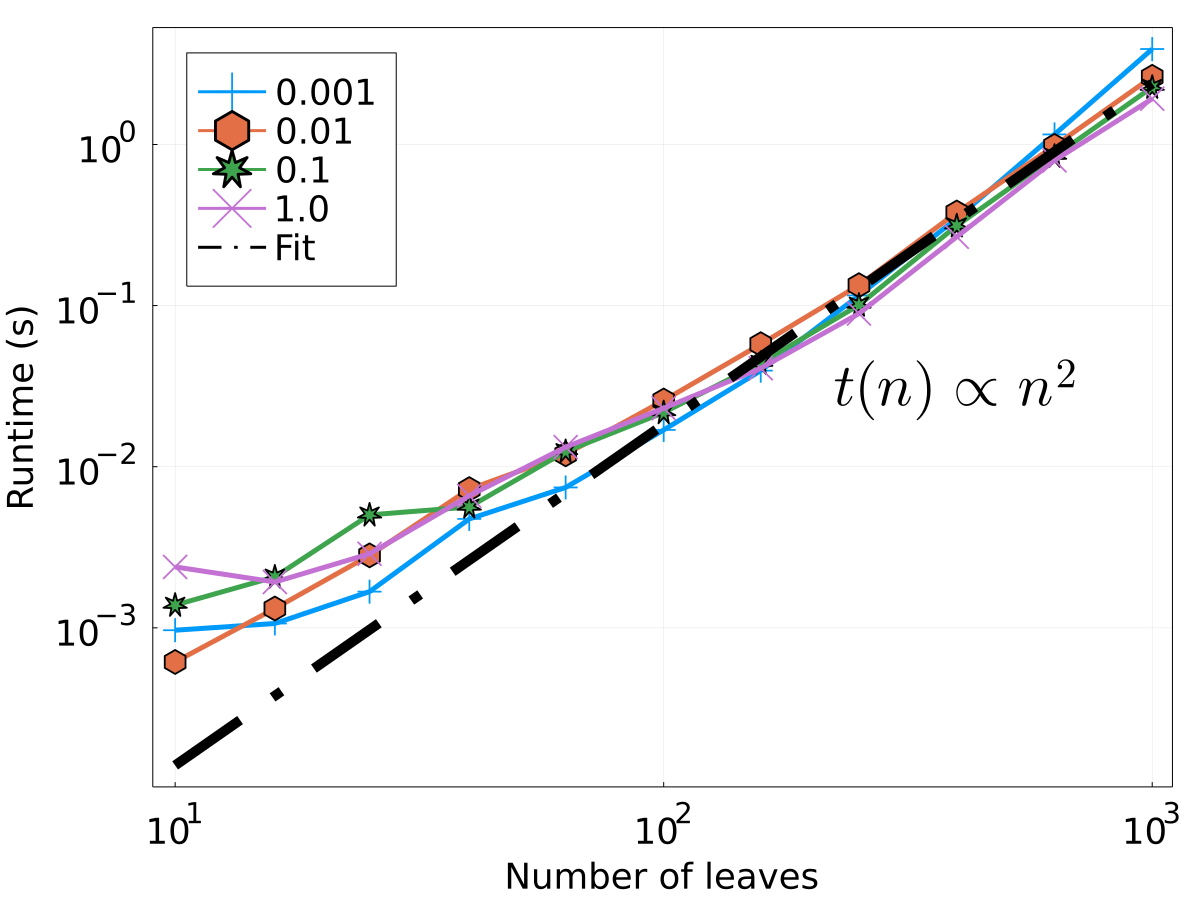

Supplement: S11 Fig — Performed on a single CPU. The linear fit is done for L > 102. As expected, runtime is quadratic in the number of leaves of the trees. (PNG) [file pcbi.1010394.s012.png]

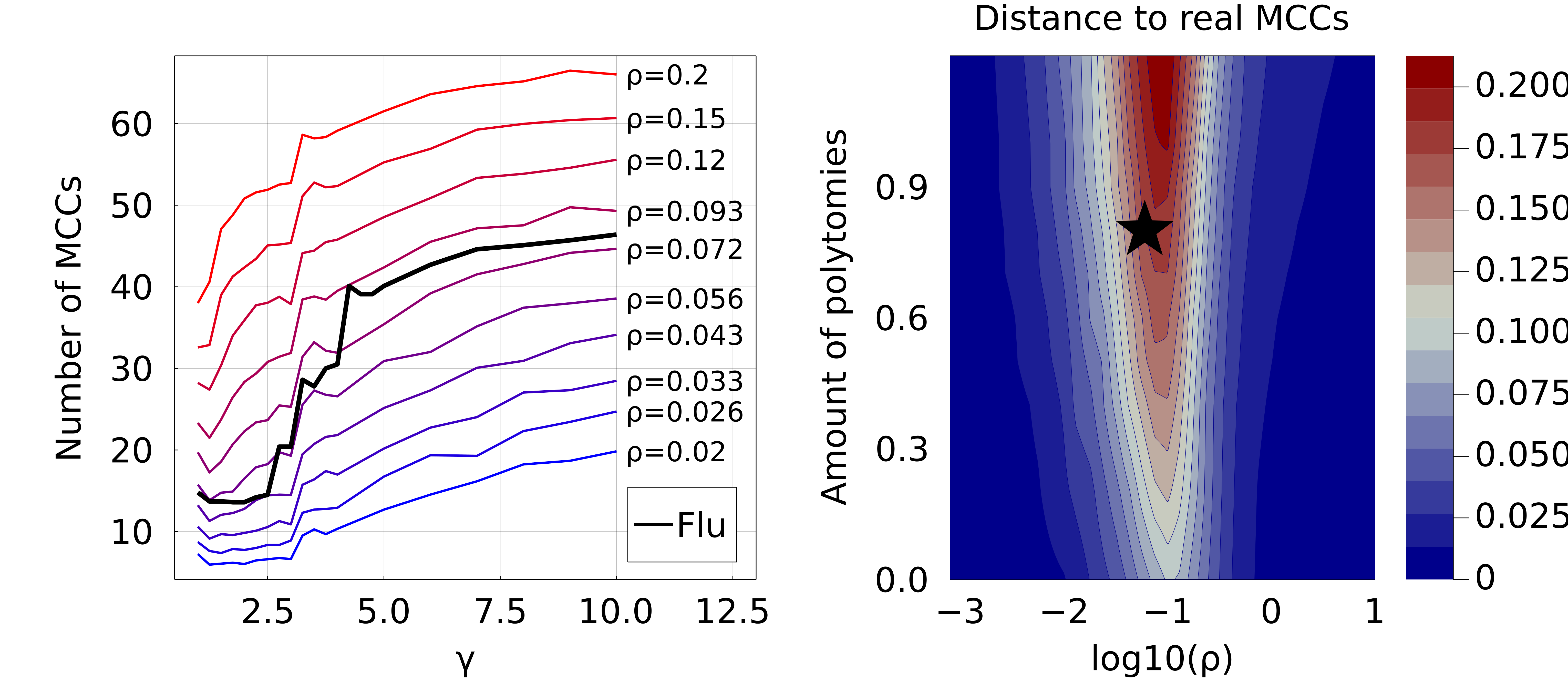

Supplement: S12 Fig — Left: Estimation of the reassortment rate of segments HA and NA in A/H3N2 influenza. Colored lines show the number of MCCs inferred by γ-methods as a function of γ. Colors going from blue to red correspond to increasing values of ρ. The black line shows the same quantity for the influenza trees. The curve corresponding to influenza lies between the values 0.043 and 0.093. Right: VI distance of inferred MCC to the real ones for simulated data and γ = 2, as a function of the reassortment rate ρ and the amount of polytomies in the trees. The star corresponds to the estimated position of A/H3N2 influenza (HA/NA segments, sequences from the same epidemiological season). (PNG) [file pcbi.1010394.s013.png]

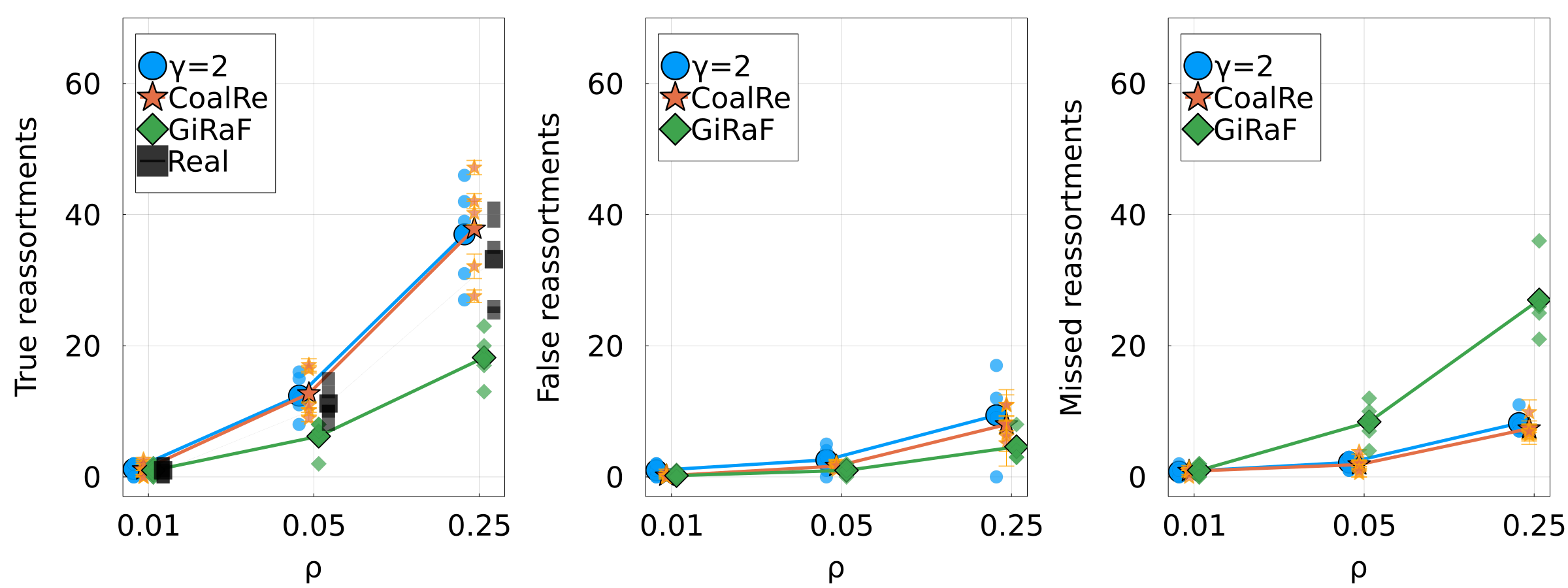

Supplement: S13 Fig — This results in reconstructed trees with more polytomies. Left: number of true reassortments, Center: number of false reassortments, and Right: number of missed reassortments, for all three methods (Treeknit, GiRaF, CoalRe). Compared to better resolved trees (Fig 5), GiRaF and Coalre infer more false reassortments while Treeknit has more missed reassortments. (PNG) [file pcbi.1010394.s014.png]

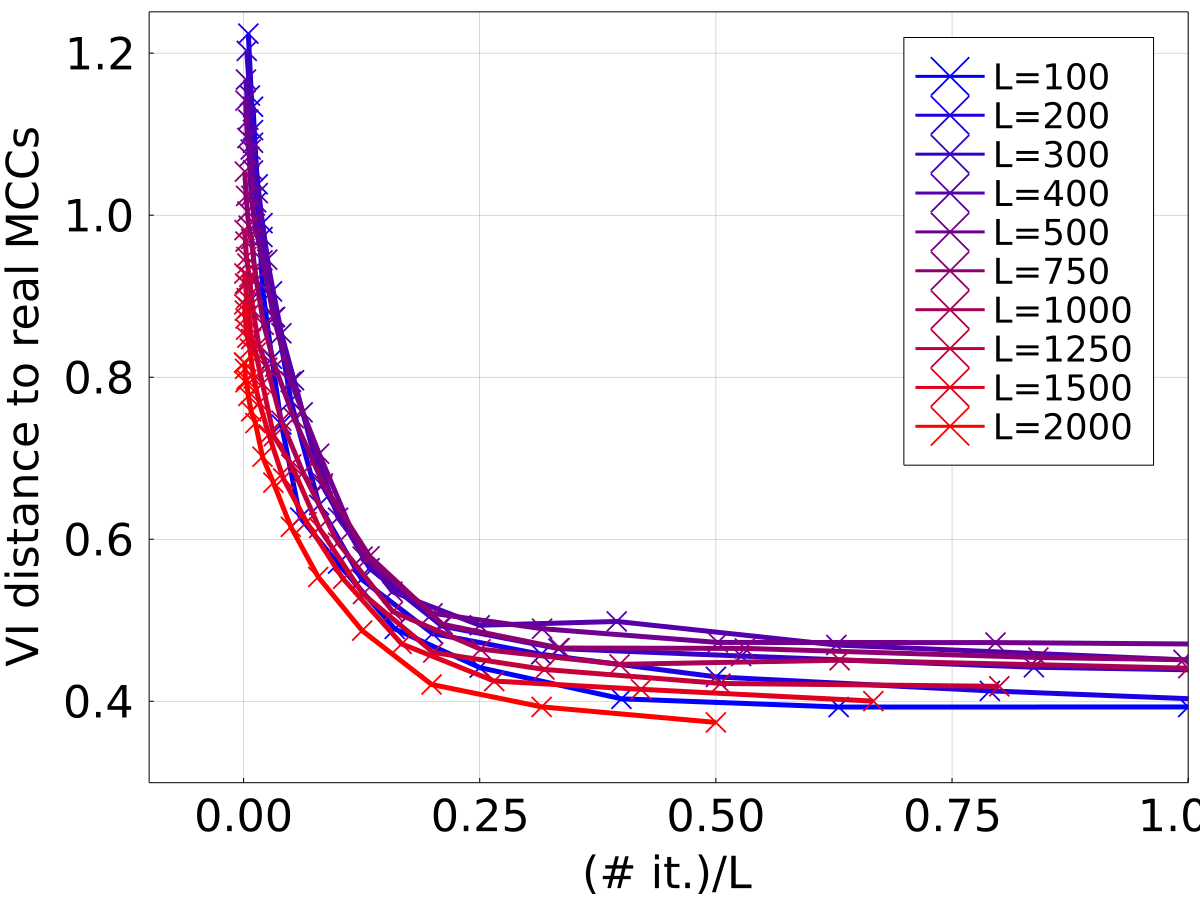

Supplement: S14 Fig — The rhythm of convergence is the same for all curves, indicating that the number of iterations needed to reach convergence should be proportional to L. (PNG) [file pcbi.1010394.s015.png]

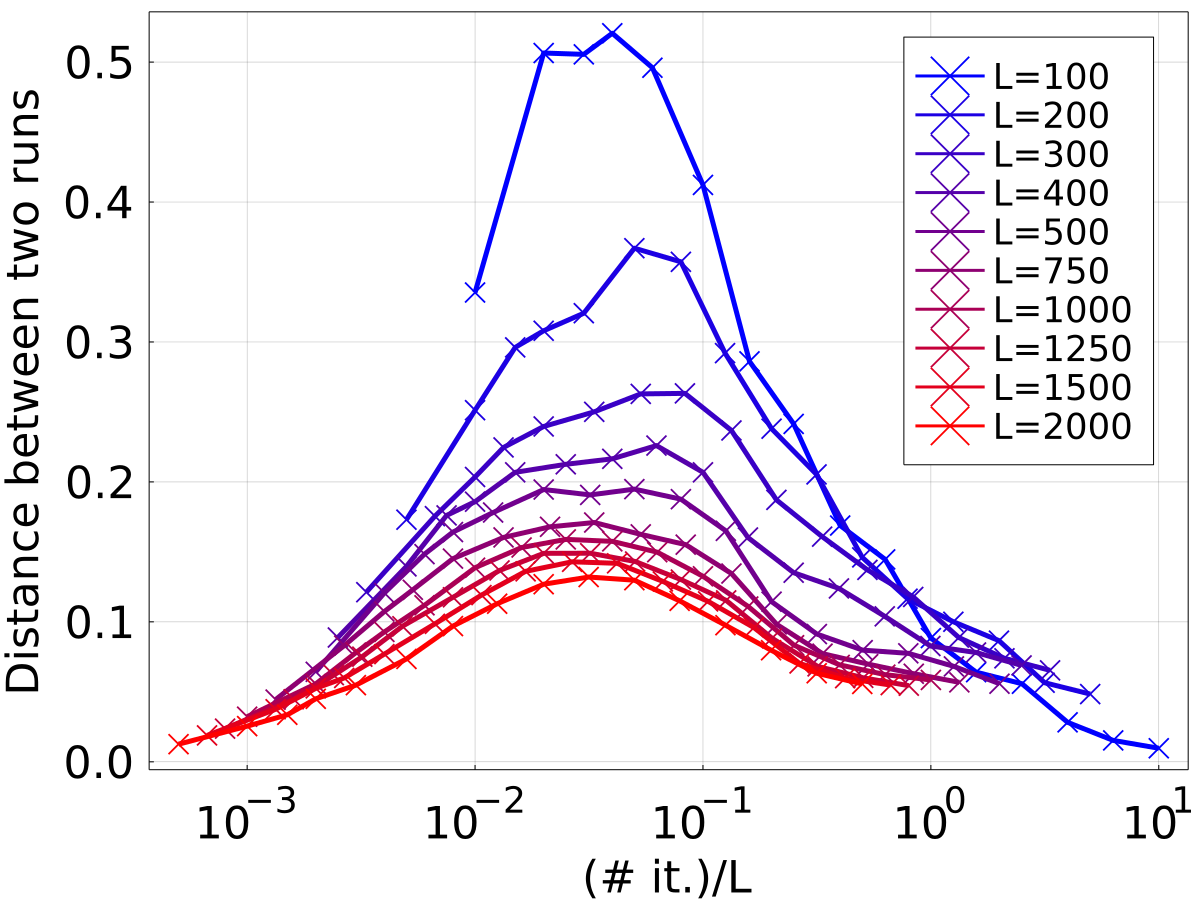

Supplement: S15 Fig — For a very low number of iterations, results are close to the naive MCCs, which are the starting point of the optimization. The distance between two runs is maximal for an intermediate number of iterations, and vanishes again as the optimization converges. (PNG) [file pcbi.1010394.s016.png]

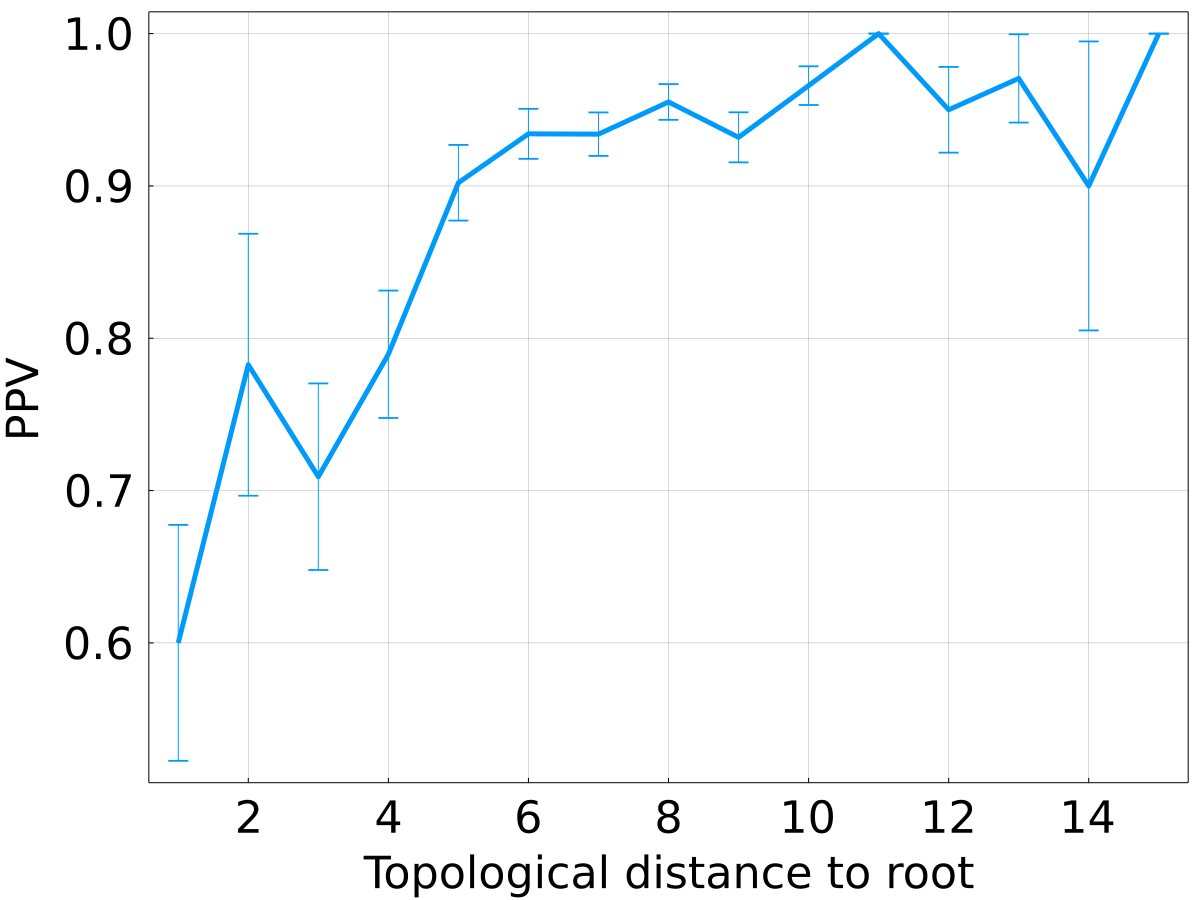

Supplement: S16 Fig — Reassortments inferred close to the root, i.e. distance ≲ 4, are more often wrong. A natural explanation for this is that errors made at an early stage of the algorithm will propagate back for the rest of the inference. Additionally, a reassortment close to the leaves can result in a large number of incompatibilities, as it can “move” a clade to a very different part of the tree. Close to the root, there are few lineages left, and thus reassortments have a weaker topological signature. (PNG) [file pcbi.1010394.s017.png]
